# A Digital Mechanistic Workflow for Predicting Solvent-Mediated Crystal Morphology: The α and β Forms of l-Glutamic Acid

**DOI:** 10.1021/acs.cgd.1c01490

**Published:** 2022-04-11

**Authors:** Thomas D. Turner, Neil Dawson, Martin Edwards, Jonathan H. Pickering, Robert B. Hammond, Robert Docherty, Kevin J. Roberts

**Affiliations:** † Centre for the Digital Design of Drug Products, School of Chemical and Process Engineering, University of Leeds, Woodhouse Lane, Leeds, LS2 9JT, U.K.; ‡ Pfizer R&D Ltd, Ramsgate Road, Sandwich, Kent CT13 9NJ, U.K.; § Britest Limited, Keckwick Lane, Daresbury, Warrington WA4 4FS, U.K.

## Abstract

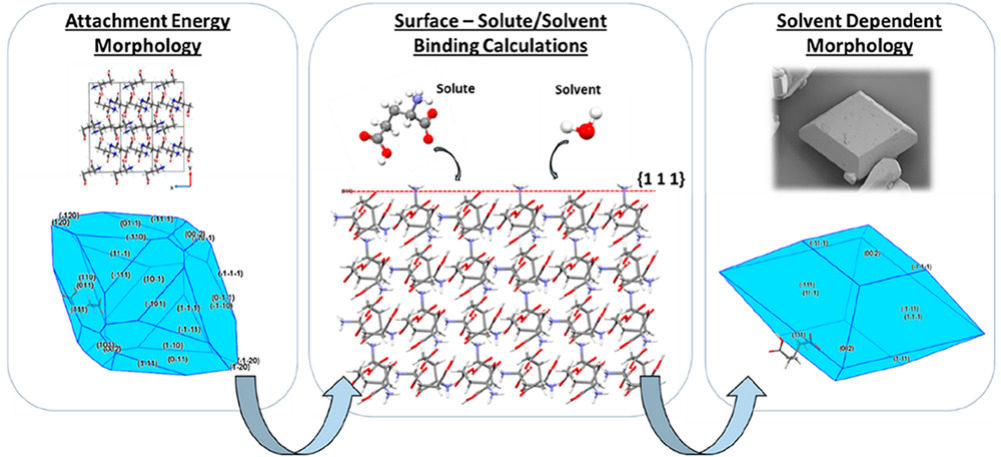

The solvent-mediated crystal morphologies of the α and β polymorphic forms of l-glutamic acid are presented. This work applies a digital mechanistically based workflow that encompasses calculation of the crystal lattice energy and its constituent intermolecular synthons, their interaction energies, and their key role in understanding and predicting crystal morphology as well as assessing the surface chemistry, topology, and solvent binding on crystal habit growth surfaces. Through a comparison between the contrasting morphologies of the conformational polymorphs of l-glutamic acid, this approach highlights how the interfacial chemistry of organic crystalline materials and their inherent anisotropic interactions with their solvation environments direct their crystal habit with potential impact on their further downstream processing behavior.

## Abstract 2

A predictive model is presented for the solvent-dependent morphology of organic materials from their root crystallographic structures through mechanistically based workflows encompassing solvent binding, surface interaction energies, and surface rugosity.

## 1 Introduction

The crystallization of organic materials forms a key step within the industrial sector where it is utilized as a common, energy-efficient methodology for the purification and isolation of high-value compounds such as active pharmaceutical ingredients (APIs) and other fine chemical products. The inherent molecular anisotropy and particle properties of these materials can have a direct impact on both product quality and downstream processing such as flowability, compactability, and bioavailability.^1,2^ The ability to control the characteristics of macroscopic crystalline particles through the rational design of the crystallization process would be of great potential importance to the industrial sector, particularly in terms of reducing bottlenecks in both R&D and manufacturing stages when developing and producing new advanced pharmaceutical products.

The physicochemical and mechanical properties of crystalline materials are governed by their intermolecular interactions (supramolecular synthons) within the solid-state (intrinsic synthons) and also, importantly, when terminated at the surfaces of specific crystal habit planes {*hkl*} (extrinsic synthons), which together characterize the surface chemistry of the crystal particle.^3^ Knowledge regarding the extrinsic synthons is also important when considering the balance between intermolecular interactions associated with solute and solvent binding at the crystal surfaces. These are associated with solute adsorption and desolvation, respectively, during the crystal growth process, and this balance ultimately directs the overall shape of the crystals and hence, through this, its overall surface properties. Many organic materials have been studied in recent years to understand the role of solvent and impurity binding at crystal surfaces in relation to their role in directing the external morphology of those materials.^4−6^ Improvements in the ability to predict crystal morphology from equilibrium methods by considering solution supersaturation and solvent/impurity binding energies have been demonstrated for a range of organic compounds crystallizing from solution environments using crystalographically-based attachment energy methods,^7−13^ including benzophenone,^14^ aspirin,^15^ and ibuprofen.^11^ Grid-based intermolecular systematic search methods have also been used to predict a range of interfacial properties such as surface wetting,^5^ API–excipient interactions,^16,17^ and solid-form salt screening.^18^

More detailed studies have made use of molecular dynamics^19^ (MD) methodologies to predict the nucleation, growth, and dissolution processes. In crystal morphology prediction, MD has revealed, e.g., detailed information concerning mechanistic and thermodynamic aspects of the growth process^20,21^ at the molecular level. However, these studies have been focused predominantly on simple organic molecules such as urea^6,22−25^ and glycine,^26−28^ this reflecting the increased computational times required for MD simulations. Kinetic Monte Carlo (kMC) methods^29^ have also been used to address some of the limitations of MD methods, and these have enabled simulations of crystal growth and dissolution processes closer to the mesoscale.^24,30,31^ kMC methods^32^ have also been coupled to MD to gain finer molecular-scale insight into the interfacial structures present at the crystal/solution interface, through calculation of the thermodynamic parameters associated with the growth process.^33^ This coupled approach has also been used to study relative crystal growth rates relating these to surface defects on the crystal faces.^34^ A useful measure of growth interfacial stability is the alpha factor^35^ which can be used to correlate interfacial stability with measured growth rates and mechanisms. Such approaches are particularly useful when characterizing crystals displaying anisotropic growth morphologies, such as needles and thin plates, where the relative growth rates of the dominant morphological forms in 3D can differ significantly.^36^

In this paper, attachment energy and grid-based systematic search methods are combined to predict the solvent-dependent morphologies of the monotropically related α and β polymorphic forms of l-glutamic acid (L-GA), providing a novel application to a polymorphic organic material which exhibits two distinct crystal habits. This is a comparatively well-studied system as evidenced by previous research regarding its solubility and nucleation kinetics,^37−40^ morphological variation,^41−44^ and phase transformation behavior,^45−49^ and hence provides a useful methodological case study system. In this work, the solid-state intermolecular interaction energies are characterized with the growth-promoting extrinsic synthons identified and cross-correlated with the surface-specific chemistry and topology of the crystal habit plane surfaces. This forms a platform for an integrated model and workflow for morphological simulation in which the attachment energy methods have been modified to take into account the solute/solvent binding energy balances as a function of the materials’ crystal habit planes as calculated using grid-based systematic search methods. Several mechanistic models have been encompassed within this workflow, and these are reviewed and discussed with respect to the observed crystal morphologies for these two polymorphic forms.

## 2 Materials and Methods

**Figure 1:**
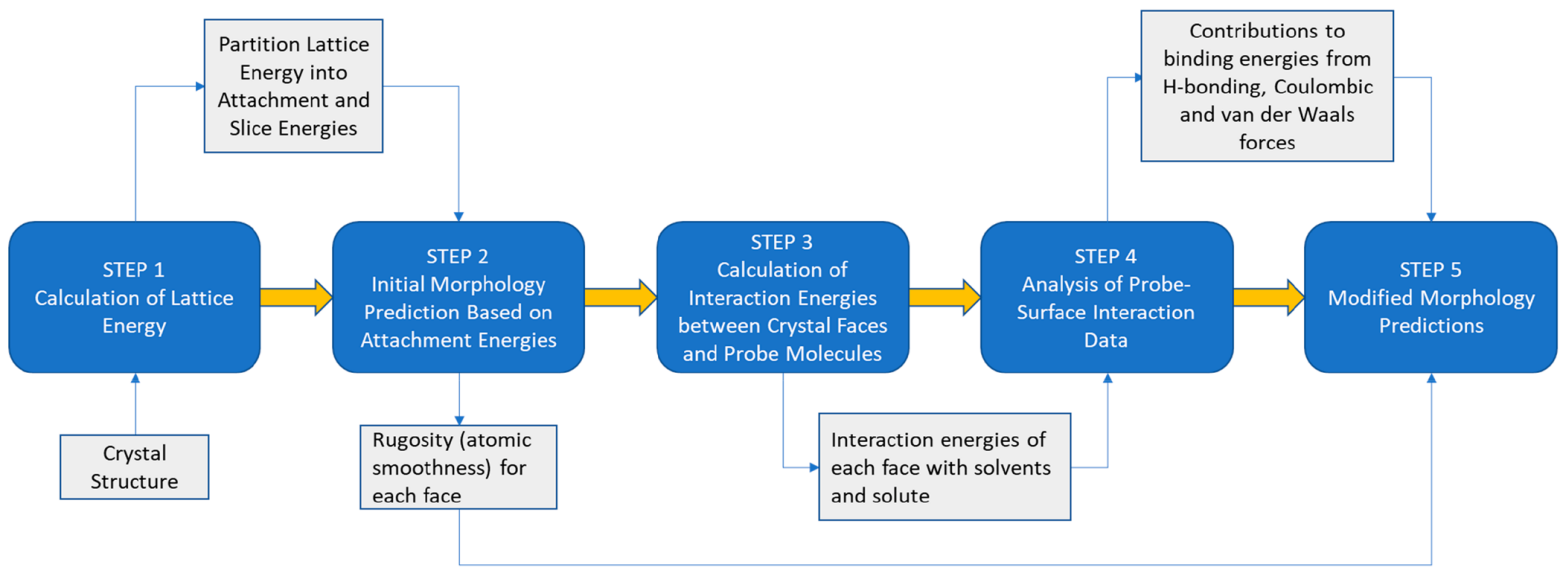
High-level, five-step process workflow for the prediction of solvent-dependent particle morphology using molecular and crystallographic modeling tools.

Figure 1 provides a high-level process flow diagram for solvent-dependent morphology prediction using the molecular and crystallographic simulation tools used in this work. These highlight the basic inputs and outputs for the main steps encompassed within the overall simulation workflow. A more comprehensive guide to the workflow is provided within Supporting Information S1.

### 2.1 Materials

l-Glutamic acid C_5_H_9_NO_4_, molecular weight: 147.13, Reagent Plus ≥99% was used as supplied by Sigma-Aldrich. Deionized water was used for recrystallization experiments.

### 2.2 Preparation of l-Glutamic Acid α and β Forms

l-Glutamic acid was recrystallized to prepare the two polymorphic forms, α and β, using a HEL Autolab 0.5L jacketed vessel with temperature control provided through a Julabo F32 recirculation chiller with a PT100 thermocouple to record the reactor temperature. The contents of the vessel were agitated at a constant stirring rate of 200 rpm with a three-blade pitched impeller. To recrystallize the metastable α form, a solution of l-glutamic acid in deionized water at a concentration of 30 g/kg was prepared in the reactor. This was then subjected to a cooling cycle from 25 °C to a holding temperature of 90 °C for 1 h to allow full dissolution of the solids. The solution was then cooled at 0.7 °C/min to a lower holding temperature of 5 °C, where the recrystallized solids were isolated using vacuum filtration and dried in an oven at 40 °C. To recrystallize the stable β form, this methodology was repeated but at an increased solution concentration of 50 g/kg with the cooling rate decreased to 0.1 °C/min.

### 2.3 Scanning Electron Microscopy

Samples were prepared for scanning electron microscopy by adhering ∼1 mg of powder from each specimen onto adhesive tabs placed on separate 12.5 mm diameter aluminum pin stubs. Excess powder was removed by tapping the stubs sharply and then gently blowing loose particles off with a jet of particle-free compressed gas. The specimen stubs prepared were sputter coated with a thin (approximately 10 nm) deposit of platinum using a Quorum Q150TS coating unit operated at 20 mA for 1 min using argon gas. The specimens were examined using a Carl Zeiss SMT SUPRA 40VP field emission scanning electron microscope (FE-SEM). The FE-SEM was operated at a high vacuum with an accelerating voltage of 3 kV and a specimen working distance of 12 mm. Secondary electron images were recorded at magnifications of 50× and 200×.

### 2.4 Morphological Modeling: Synthon Strengths, Lattice Energy, and Baseline Morphology

Molecular modeling of the l-glutamic acid polymorphs was carried out using Materials Studio,^50,51^ Habit98,^52,53^ and the Cambridge Crystallographic Data Centre’s (CCDC^54^) Mercury software. The crystal structures of the α (ref code LGLUAC02^55^) and β (ref code LGLUAC01^56^) polymorphs, as obtained from the CCDC database, were optimized using the Forcite module of Materials Studio, where the unit cell parameters were allowed to relax with the motion groups being held rigid. The force field used was Dreiding,^57^ and atomic charges were derived using the Gasteiger^58,59^ approach.

The intermolecular interactions which contribute to the stabilization of the two lattice structures were analyzed using Habit98^52,53^ utilizing an atom–atom approach.^60^ Further to this, the lattice, *E*_latt_, slice, *E*_sl_, and attachment, *E*_att_ energies were calculated from the strengths of these intermolecular interactions, using the Momany^61^ interatomic potential set together with partial atomic charges calculated using Mopac.^62^ The lattice energy was calculated by constructing a network of unit cells and calculating the intermolecular interactions at increasing sphere sizes expanding from a central molecule. The calculated lattice energy was cross-correlated to the known experimental sublimation enthalpy, *ΔH*_s_, of the β polymorph^63,64^ through eq 1 in order to assess the suitability of the potential, and where *R* is the ideal gas constant, *T* is the absolute temperature, and *ΔE*_pt_ is the proton transfer energy reflecting the zwitterionic nature of l-glutamic acid in its solid-state form.
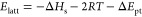
1

The calculated lattice energy of the two polymorphs was further partitioned onto the atoms of the molecules within the asymmetric unit to provide an analysis of the contribution of the molecular functionalities to the stabilization of the crystal lattice. The overall convergence of the lattice energy calculation was assessed by 1 Å stepwise calculations between 5 and 25 Å of the overall calculation sphere.

To understand the important synthons associated with crystal growth, the calculated synthons were partitioned between the intrinsic (*E*_slice_) synthons that were fully saturated within the surface growth terraces and the extrinsic synthons which were surface terminated (*E*_att_); this is highlighted in eq 2.^65^
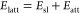
2

The slice energy (*E*_slice_) was used to describe the anisotropy of a specific *hkl* plane according to eq 3,^66,67^ where the anisotropy factor, ε_*hkl*_, can be taken as a measure of the surface saturation of synthons for a defined crystal habit surface upon termination of that surface.
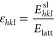
3

The calculated values of *E*_att_ allowed scaling of the relative growth rates of the crystal surfaces to predict a particle morphology using a Wulff plot^68,69^ as implemented in CCDC’s Mercury.^54^

The α factors were calculated using the approximation given in eq 4,^35^
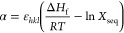
4where *ΔH*_f_ is the enthalpy of fusion, and *X*_seq_ is the mole fraction of the solute at a relevant supersaturation and temperature for crystal growth of the solute system.

### 2.5 Calculation of the Solute/Solvent Binding to the Crystal Habit Faces

The rigid-body intermolecular interaction energies for the habit surfaces of LGA interacting with LGA (solute phase) and H_2_O (solvent phase) probe molecules were predicted using the Systematic Search method.^15−17,70,71^ The interaction energies were calculated using an atom–atom summation method between the probe molecules and the molecules in the slab of the unrelaxed crystal surface. The probe molecules were moved to various grid points in 0.2 Å steps, covering the crystal surface, and at each grid position the probe molecules were rotated through three Euler angles in 30° steps to cover the rotational degrees of freedom of the molecule close to the surface. At each grid point and its subsequent rotational steps, the interaction energy between the probe molecules with the surface was calculated using the Momany^61^ empirical force field together with atomic charges as calculated using the Gasteiger^58,59^ method. A more detailed description of the surface searching methodology is provided in the Supporting Information S1.

### 2.6 Integration of Attachment Energy Models

The calculated solute and solvent binding energies were used to adjust the calculated surface attachment energies using three different functional forms and through this, modified solvent-dependent surface attachment energies were calculated.

The first functional form used the relationship developed by Hammond et al.,^15,72^ which yielded eq 5, where *U*_*εhkl*_ is the modified attachment energy of a specific *hkl* plane, and *U*_solute_ and *U*_solvent_ are the strongest interaction energies for the surface–solute and surface–solvent binding as calculated using the systematic search methodology, respectively.
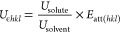
5

The second functional form involves a modification of eq 5 to allow the atomic scale topology of the crystal surface to be factored into the calculation. In this case, a surface rugosity factor the plane rugosity, *R*_g_, is included as a fraction of the plane with the lowest rugosity, *R*_g min_, and is provided in eq 6. *R*_g_ was calculated as the root-mean-square deviation of the displacement of the atomic centers of the molecules present in a crystal surface of a single *d*-spacing thickness and is a useful representation of surface “roughness” at the molecular scale.
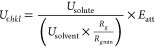
6

The final functional form encompasses the surface entropy or α factor as defined by Jackson (1958), which provides an indication regarding the “reactivity” of the crystal surface, notably its propensity to bind solute and solvent molecules. Equation 7 takes this into account in terms of solvent binding on the various crystal surfaces.^35,67,73−75^ This allows a representation as to the ease of solute attachment to a growing surface based on the intermolecular extrinsic synthons bonding terminated at the crystal habit surface.
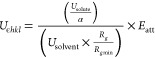
7

## 3 Results and Discussion

### 3.1 Crystal Structures and Associated Molecular and Crystal Chemistry

**Figure 2:**
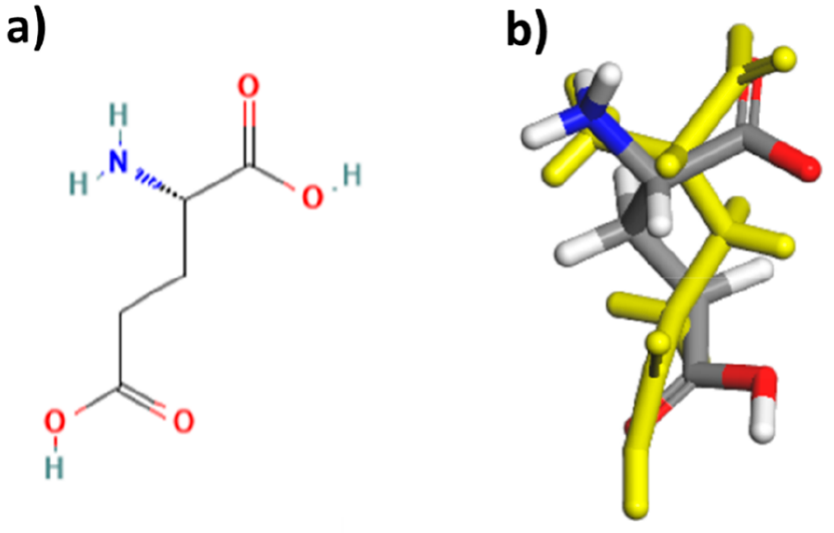
(a) l-gGlutamic acid molecular diagram, (b) overlay of the two l-glutamic acid conformers associated with the α and β polymorphic forms; the α conformer is colored by the atom type, and the β form is colored yellow for comparison.

**Figure 3:**
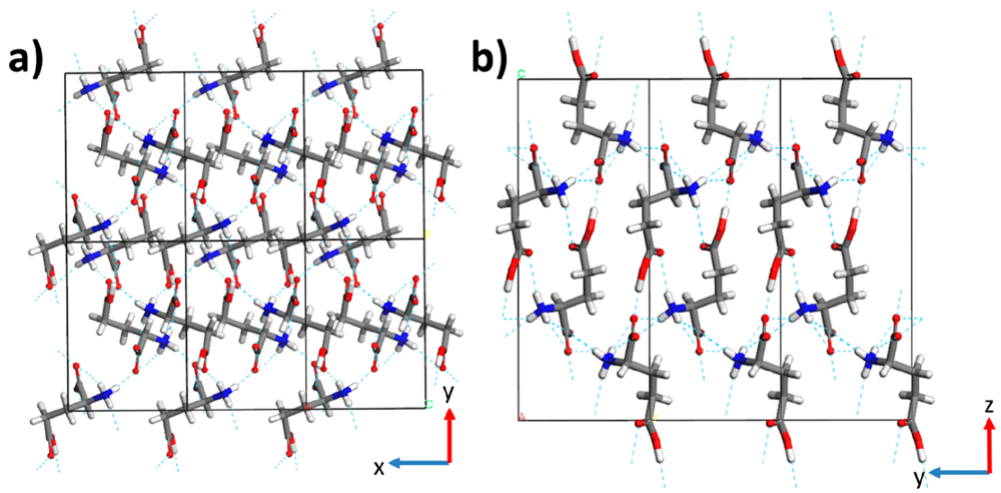
Crystal structure packing diagrams of (a) the α polymorph viewed down the *c* axis and (b) the β polymorph viewed down the *a* axis.

l-Glutamic acid has two polymorphs, α and β, which are monotropically related, and the material is zwitterionic in both the crystal structure and in solution. The two forms are conformational polymorphs, where the stable β conformer adopts a slightly more planar conformation of the carbon backbone when compared to the metastable α conformer; these differences are highlighted in Figure 2. The two polymorphs of LGA both crystallize in an orthorhombic crystal structure in a *P*2_1_2_1_2_1_ space group, and the crystal intermolecular packing structures of both polymorphs are shown in Figure 3.

The intermolecular packing for the α polymorph encompasses a three-membered H-bonding ring structure which is extended down the *a*-axis by CO–HN interactions as shown in Figure 3a. Similarly, the *b*-axis is characterized by a separate three-membered H-bonding ring which is formed through CO–HN interactions. The *c*-axis for the α form has an alternating double ring structure which is linked though ammonium–carboxylate, ammonium–carboxyl, and carboxylate–hydroxyl interactions.

In contrast, the *a*-axis of the β polymorph is characterized by two types of carboxylate–ammonium Coulombic interactions: the first a zigzag unbroken chain of alternating NH_3_^+^–CO_2_^–^ and CO_2_^–^–NH_3_^+^ interactions, and the second involves a single carboxylate–ammonium interaction in unbroken chains. The *b*-axis is characterized by two types of ammonium–carboxylate Coulombic interactions which form unbroken chains in this lattice direction, highlighted in Figure 3b. The long crystallographic *c*-axis of the β structure contains OH–O H-bonds which form an extended unbroken chain in this lattice direction.

A comparison between the bulk crystal chemistry of the α and β forms highlights that both polymorphs display an extensive network of Coulombic and H-bonding interactions in all three principle lattice directions, and hence overall the polymorphs can be classified as three-dimensionally hydrogen-bonded materials.

### 3.2 Lattice Energies and their Convergence

**Figure 4:**
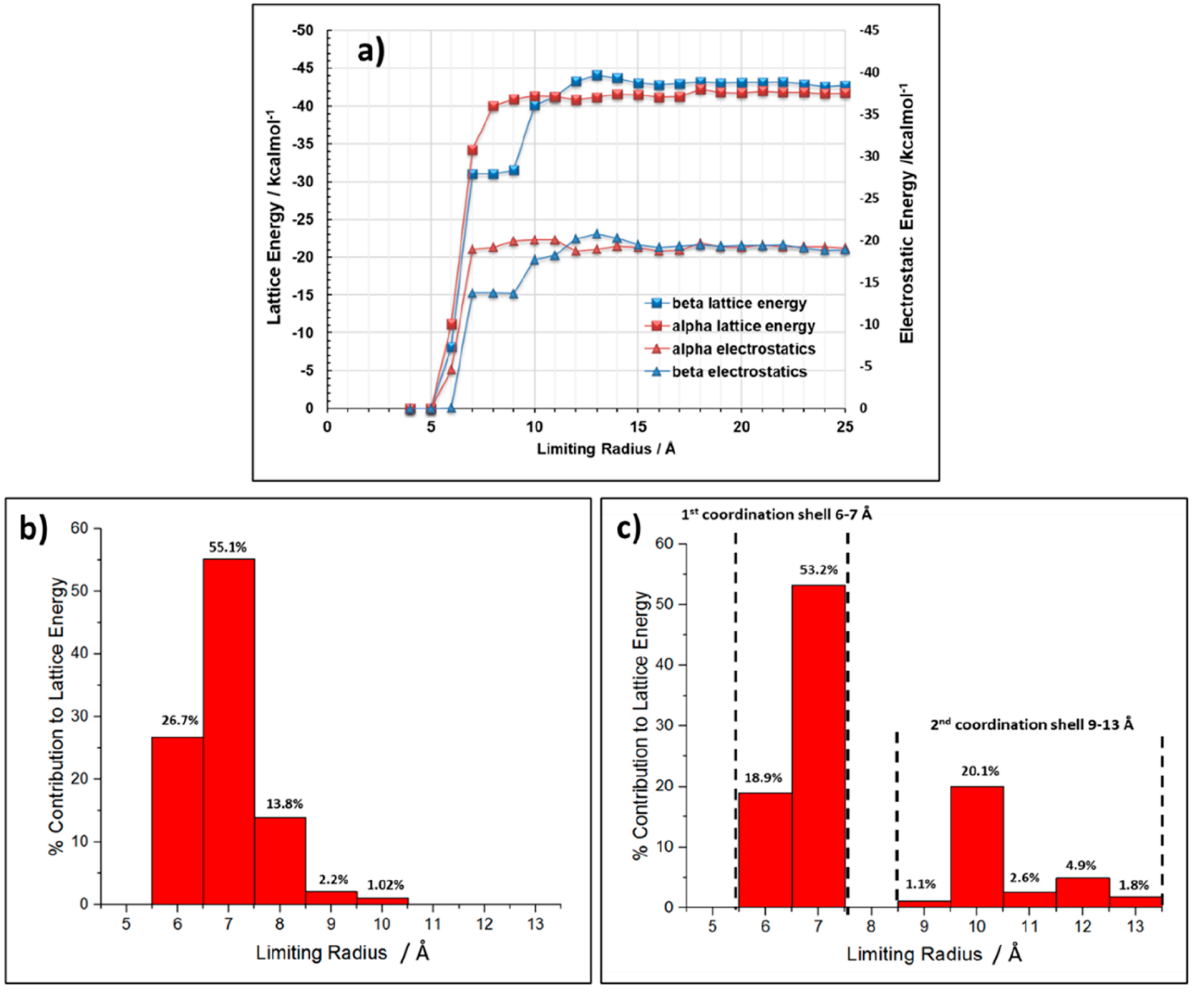
(a) Convergence of calculated lattice energy and the associated contribution of the electrostatics to overall lattice energy as a function of limiting radius (b) and (c) radial distribution plots showing the percentage contribution to the lattice energy for each discrete addition of radius to the calculation sphere for (b) α polymorph and (c) β polymorph.

The crystallographic data for the two polymorphs are summarized in Table 1. The calculated lattice energies were found to be −41.83 kcal mol^–1^ and −43.03 kcal mol^–1^ for the α and the β polymorphic forms, respectively. This is in good agreement with the literature values for previously calculated lattice energies of the two polymorphs^76^ and also with experimental sublimation enthalpy data.^63,64,77^ Examination of the convergence of the lattice energy summation, shown in Figure 4a, reveals that the Coulombic interactions contribute ∼50% of the total lattice energy for both polymorphs. It was found that the α form lattice energy converges at a lower limiting radius, whereas the β form lattice energy converges over two coordination shells as shown in Figure 4. Figure 4b indicates that 81.8% of the total α form lattice energy was added after 7 Å. By comparison, Figure 4c shows that 72.1% of the β form lattice energy was added after 7 Å, and the remainder is in a second coordination shell spanning intermolecular interactions within 9–13 Å, with full convergence taking place at a larger limiting radii. Overall, the data are consistent with the formation of smaller stable molecular clusters for the α form in comparison with the β form. This prediction supports the hypothesis that the α form in both pre- and postnucleation stages would be more stable than that of the β form, in good agreement with previously calculated cluster energies as a function of size.^78^ In relation to crystallization conditions, this would suggest that high supersaturation, i.e. small critical cluster sizes, would favor the crystallization of the eventually metastable α form with respect to the eventually stable β form and vice versa. Such behavior suggests that while short-range intermolecular interactions would appear to favor the formation of the α form, as the clusters grow and develop into macroscopic crystals the longer-range intermolecular packing forces tend to play the more dominant role, hence enabling the transformation of the α form to the β form for which the latter has a higher density and lower void space when compared to the α form.

**Table 1:** Crystallographic Structure Information and Calculated Lattice Energy for the Two Polymorphs of l-Glutamic Acid

material descriptor	α	β
Refcode	LGLUAC02^55^	LGLUAC01^56^
molecular surface area (Å^2^)	152.60	151.92
molecular volume (Å^3^)	133.87	129.23
*a* (Å)	7.1777	4.9652
*b* (Å)	10.3986	6.8591
*c* (Å)	8.8996	17.8457
volume (Å^3^)	664.2486	607.7675
crystal density (g/cm^3^)	1.47	1.61
packing coefficient	0.81	0.85
void space (%)	21.7	20.0
space group	*P*2_1_2_1_2_1_	*P*2_1_2_1_2_1_
*Z*	4	4
*Z*′	1	1
*E*_cr_ (kcal mol^–1^)	–41.83	–43.03
H-bond donors	3	3
H-bond acceptors	8	8

**Figure 5:**
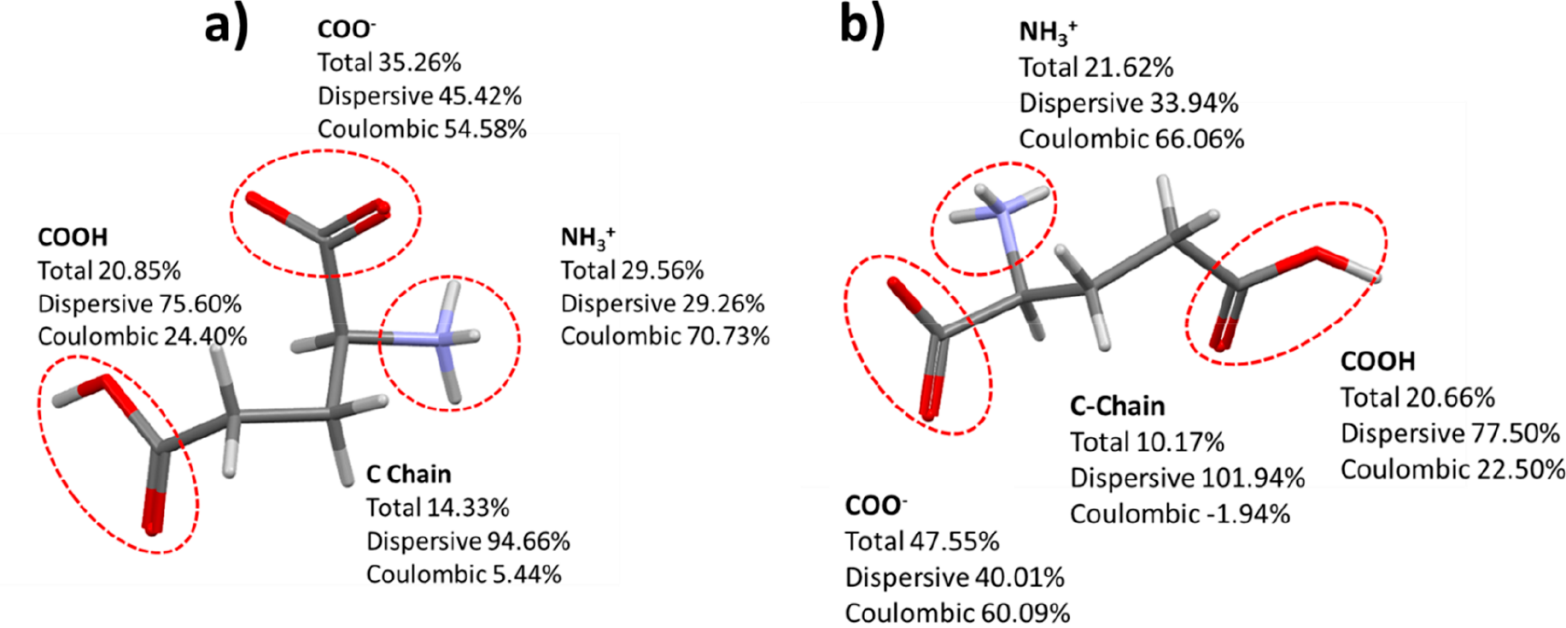
Contribution of the molecular fragments to the calculated lattice energy together with the dispersive and Coulombic component of those contributions expressed as a percentage for the asymmetric unit of the α (a) and β (b) asymmetric units.

The partitioning of the calculated lattice energies onto the different functional groups of the LGA molecule for the two polymorphs is shown in Figure 5, a and b, respectively. This reveals that the interactions between the carboxylate and ammonium ions dominate the lattice energies, contributing 64.82% in the α structure and 69.17% in the β structure highlighting the importance of these electrostatic interactions in terms of their role in stabilizing the solid-state structure for both polymorphs. Intermolecular interactions involving the aliphatic chain in LGA were found to be less important in terms of their contributions to the lattice energy, albeit its contribution was found to be ∼4% greater in the α form structure when compared to the β form structure. The latter may reflect the more planar nature of the aliphatic chain in the β form conformation with respect to the α form, which results in a degree of shielding of the carbon chain by the carboxylate and carboxylic acid functionalities. This, in the β form structure, decreases the dispersive intermolecular interactions of the carbon chain with those of its neighboring molecules.

### 3.3 Analysis of Intrinsic Synthons within the Solid-State

Table 2 summarizes the strongest intermolecular interactions for the α and β form polymorphs, highlighting the importance of the zwitterionic functional groups in terms of their contributions to the intermolecular energy. This is not a surprising result considering the contributions made by the ammonium and carboxylate groups in terms of lattice stability for both polymorphs.

**Table 2:**
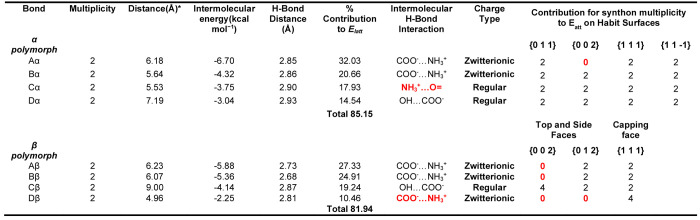
Strongest Intermolecular Interactions in the α and β LGA Structures Indicating the Strength of the Interactions, the Center of Gravity Distances between Interacting Molecules, the Contribution to the Lattice Energy, and the Specific Type or Chemistry of the Synthonic Interaction^a^

a Interactions in bold red indicate functional group differences in h-bonding synthons between the two polymorphs, together with the individual synthon contribution of multiplicity towards the total *E*_att_ for the separate observed habit surfaces of the α and β polymorphs which are highlighted in Table 3; the significant differences between habit faces for synthon multiplicity contribution are highlighted in bold red text beneath the respective habit face.

b Distance is calculated from the center of gravity of the two molecules contributing to the intermolecular interaction.

**Figure 6:**
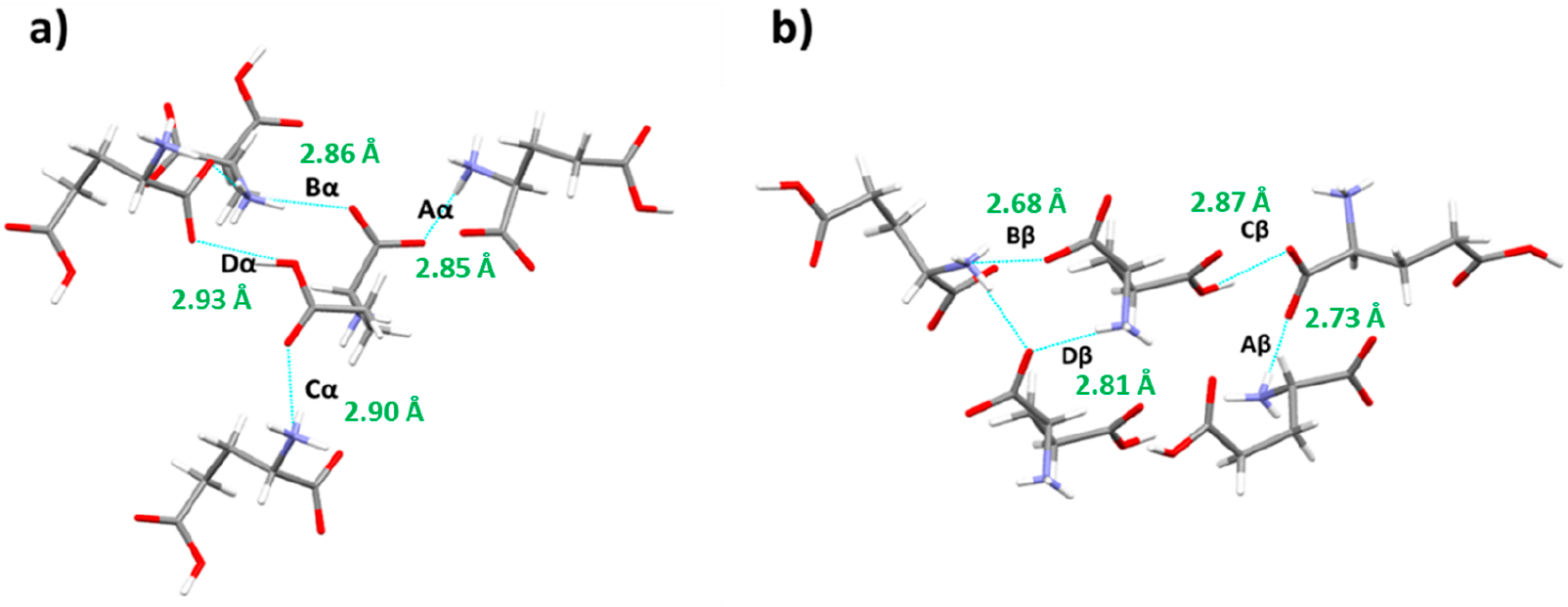
(a) l-Glutamic acid α polymorph strongest intermolecular interactions (synthons); dashed blue lines indicate specific interaction (b); strongest intermolecular interactions (synthons) contained within the structure of the β polymorph. The nomenclature used is where the letter denotes the synthon ranking as a function of total energy; i.e., A is the strongest intermolecular interaction, and D the weakest in the top four, and the Greek letter refers to the polymorph. The synthons are further detailed in Table 2, and the H-bonding interaction distance (Å) is also highlighted for each synthon.

The strongest intermolecular synthon in the α form structure, Aα, involves a very directional Coulombic interaction between the ammonium and carboxylate groups, which contributes 32.03% of the total lattice energy. While the second most important synthon, Bα, also involves strong Coulombic interaction between the ammonium and carboxylate groups, as shown in Figure 6a, this interaction is offset with respect to the electron cloud of the carboxylate group resulting in the interaction being significantly weaker in comparison to Aα with an interaction energy of −4.32 kcal mol^–1^. The Cα and Dα synthons both involve H-bond formation between the carboxylic acid group with the carboxylate and ammonium groups, respectively, both of which were found to be weaker than both the directional and offset Coulombic interactions of the Aα and Bα synthons, respectively, with Cα = −3.75 kcal mol^–1^ and Dα = −3.04 kcal mol^–1^.

In the β form structure, while the strongest intermolecular synthon, Aβ, was also found to involve a Coulombic interaction between the ammonium and carboxylate groups, this was found to be a weaker and less close-packed interaction −5.88 kcal mol^–1^; in comparison to the synthon Aα in the α form, associated with a longer interaction distance of 6.23 Å compared to 6.18 Å in the α form. Interaction Bβ was also found to be a Coulombic interaction which again was similar to the Aα and Bα interaction albeit with increased interaction distances and hence a comparatively lower interaction energy of −5.36 kcal mol^–1^ to Aβ but higher than Bα. The intermolecular synthon Cβ was found to contain a directional OH–O hydrogen bond with a stronger interaction energy of −4.4 kcal mol^–1^ compared to the similar Dα interaction in the α form structure. The Dβ synthon, consisting of a stacked Coulombic interaction between the ammonium and carboxylate functionalities, was found to be the weakest of the top four interactions within the β form structure with a calculated energy of −2.25 kcal mol^–1^.

A comparison between the crystal chemistry of the two polymorphic forms in relation to these strongest interactions reveals, in general, shorter absolute H-bond interaction distances (2.68–2.87 Å) for the β form with respect to the α form (2.85–2.93 Å), shown in Table 2. This is consistent with more efficient close packing of molecules in the β form, which is also reflected in the respective packing coefficients and densities for the β (0.85) and α (0.81) forms, as highlighted in Table 2. These differences reflect the more compact and planar molecular conformation in the β form relative to the α structure, with higher molecular surface area and volume found in α, 152.6 Å^2^ and 133.87 Å^3^ respectively when compared to β 151.92 Å^2^ and 129.23 Å^3^ respectively. Additionally, the overall energetic differences between the top four intermolecular synthons were found to be relatively similar for the two forms. Further detailed images of the four dominant intermolecular synthons for both forms are provided in S2 of the Supporting Information.

### 3.4 Prediction of Crystal Morphology Based upon Crystallographic Structure

**Figure 7:**
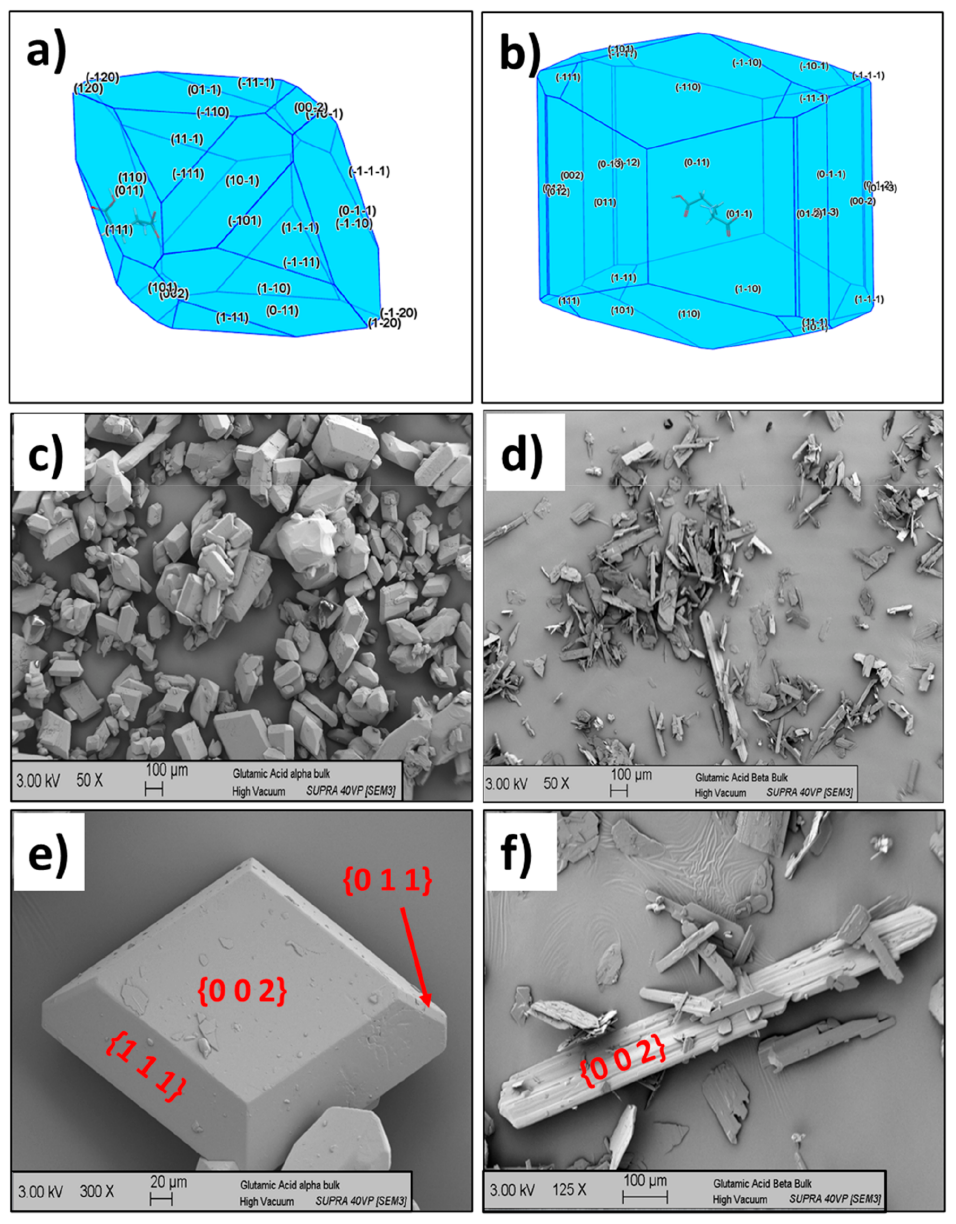
(a) Attachment energy morphology calculated for the α polymorph of l-glutamic acid, (b) attachment energy morphology calculated for the β polymorph of l-glutamic acid, (c, d) SEM images of bulk α and β crystals prepared from water solutions respectively, highlighting the poor correlation of the attachment energy model to the experimental morphology in water of the two forms, (e, f) more detailed SEM images of individual α and β form crystals, respectively, with their habit faces labeled.

The predicted crystal morphologies for LGA are provided in Figure 7 together with SEM micrographs of the experimentally observed crystals. Examination of the α form, Figure 7a reveals a prismatic type crystal where the {0 1 1} and {1 1 0} surfaces dominate the morphology. However, this is in poor agreement with the observed experimental morphology in Figure 7c, which reveal a more equant and tabular crystal habit dominated by the {0 0 2} and {1 1 1} surfaces.

The predicted *E*_att_ morphology for the β form polymorph (Figure 7b) reveals a morphology that is dominated by {0 1 1} and {1 1 0} surfaces in a prismatic crystal habit. This too does not correlate well with the observed morphologies in Figure 7d, which reveal a needle-like crystal habit, dominated by large {0 0 2} surfaces.

### 3.5 Prediction of Solvent and Solute Binding on the Crystal Habit Surfaces

#### 3.5.1 Analysis of Strongest Binding Site Energies

Table 3 shows the most favorable calculated interaction energies of water and LGA probe molecules on the predicted crystal habit surfaces for the eight (highest *d*-spacing) surfaces of the α and β form polymorphs. The habit faces of the α and β form polymorphs have been previously identified in the literature.^41,43^ The large surfaces of the prismatic α form exhibit the {1 1 1} and {0 0 2} habit planes, while the needle-like β form exhibits {1 1 1}, {0 0 2}, and {0 1 2} surfaces. These surfaces are highlighted in red in Table 3.

**Table 3:**
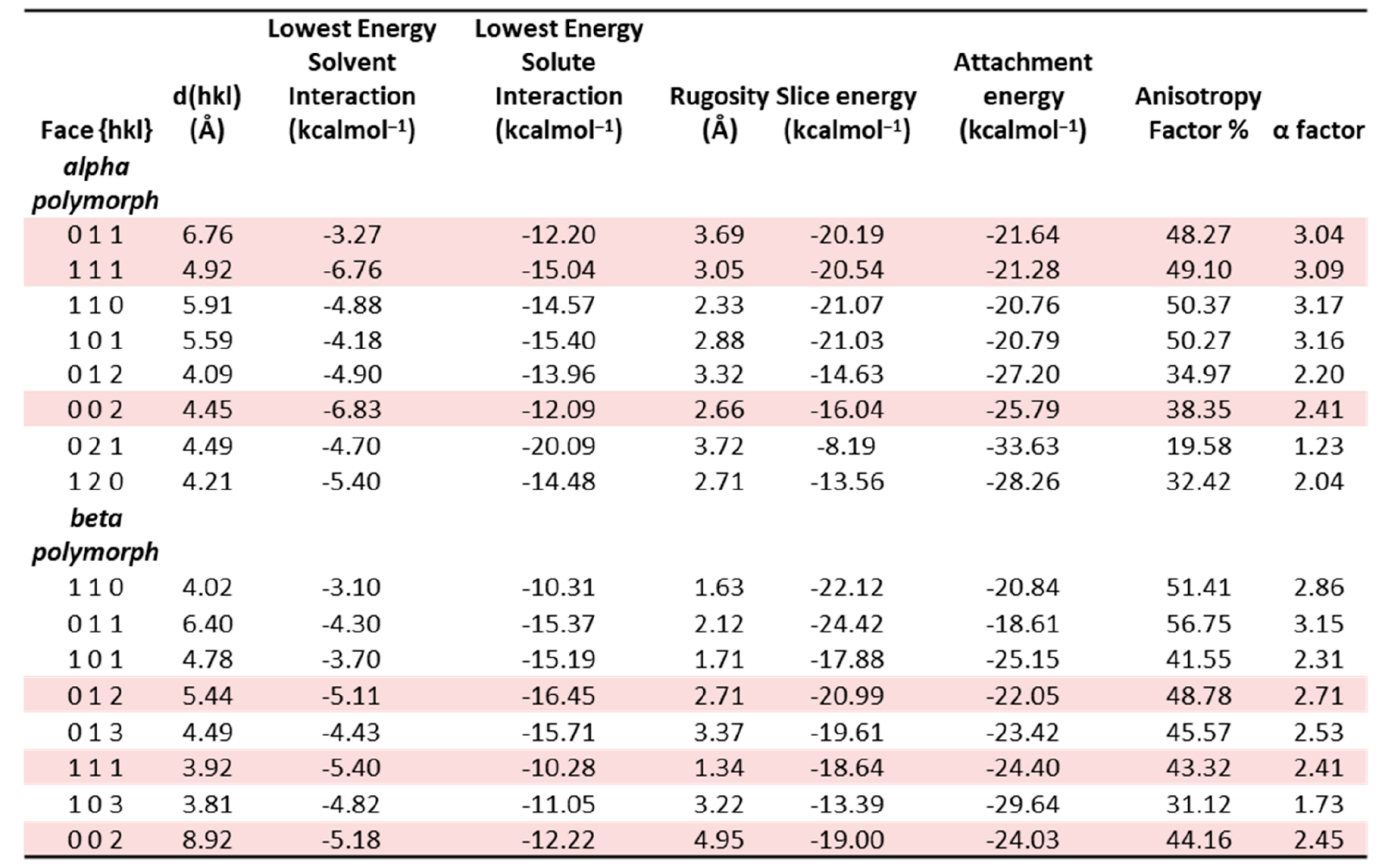
Surface Search Results for the α and β Polymorphs Highlighting the Lowest Interaction Energies of Water and Glutamic Acid Probes at the Crystal Planes from the Top Eight Largest *d*-Spacing BFDH List Together with Calculated Plane Rugosity, Slice and Attachment Energy, Surface Synthon Saturation Expressed As a Percentage of the Anisotropy Factor and the Calculated α Factors

Interestingly, these known crystal habit surfaces, in general, were found to exhibit more favorable interaction energies with the solvent probe molecule relative to the other surfaces. In particular, the {0 0 2} and {1 1 1} of the α form have strong interactions with water, −6.83 and −6.76 kcal mol^–1^ respectively. This is consistent with their desolvation being less energetically favorable in aqueous solution with respect to other faces and hence possibly reducing the effective growth rate of these surfaces, which in turn increases their surface area and morphological importance.

**Figure 8:**
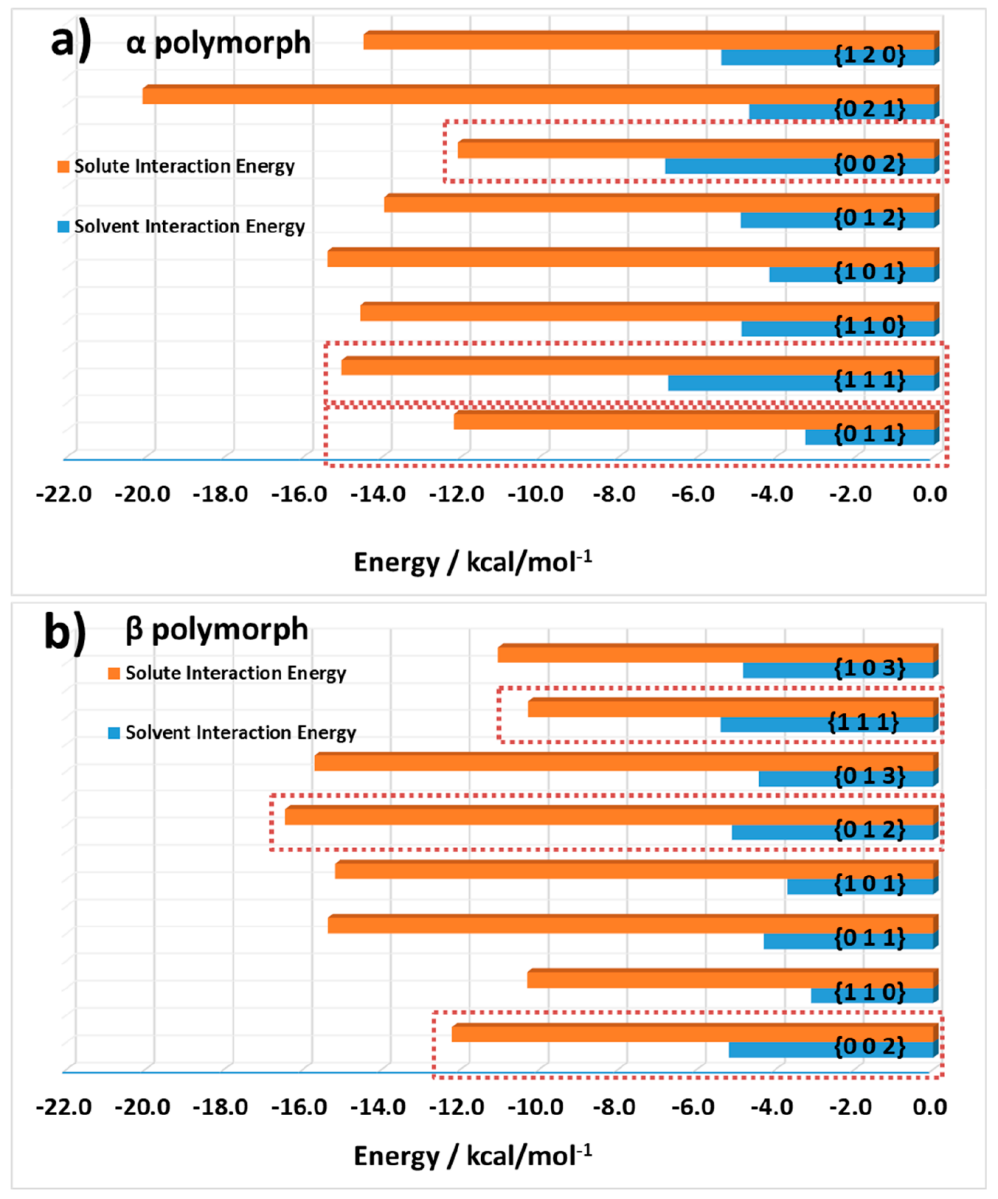
Calculated most favorable solute and solvent interaction energies at the various surfaces of the (a) α polymorph and (b) β polymorph to provide a comparison as to the ease of solute integration; the surfaces shown in red boxes indicate the surfaces present on the final particle morphology.

Examination of the data for the β form polymorph reveals a similar trend, where the strongest interaction of the water probe is on the {0 0 2}, {1 1 1}, and {0 1 2} surfaces, −5.18, −5.40, and −5.11 kcal mol^–1^ respectively. These surfaces have also been reported in the literature as the possible habit faces of the β form crystals grown from water solutions.^41,43^ Overall, this analysis highlights the importance of considering the balance between the surface adsorption of solute molecules and the desolvation of solvent molecules at the growing crystal habit planes when modeling crystal morphology under realistic recrystallization conditions. These differences between the solute and solvent interaction energies at the various surfaces are summarized in further detail in Figure 8.

#### 3.5.2 Analysis of the Interfacial Chemistry Associated with Solvent Binding

**Figure 9:**
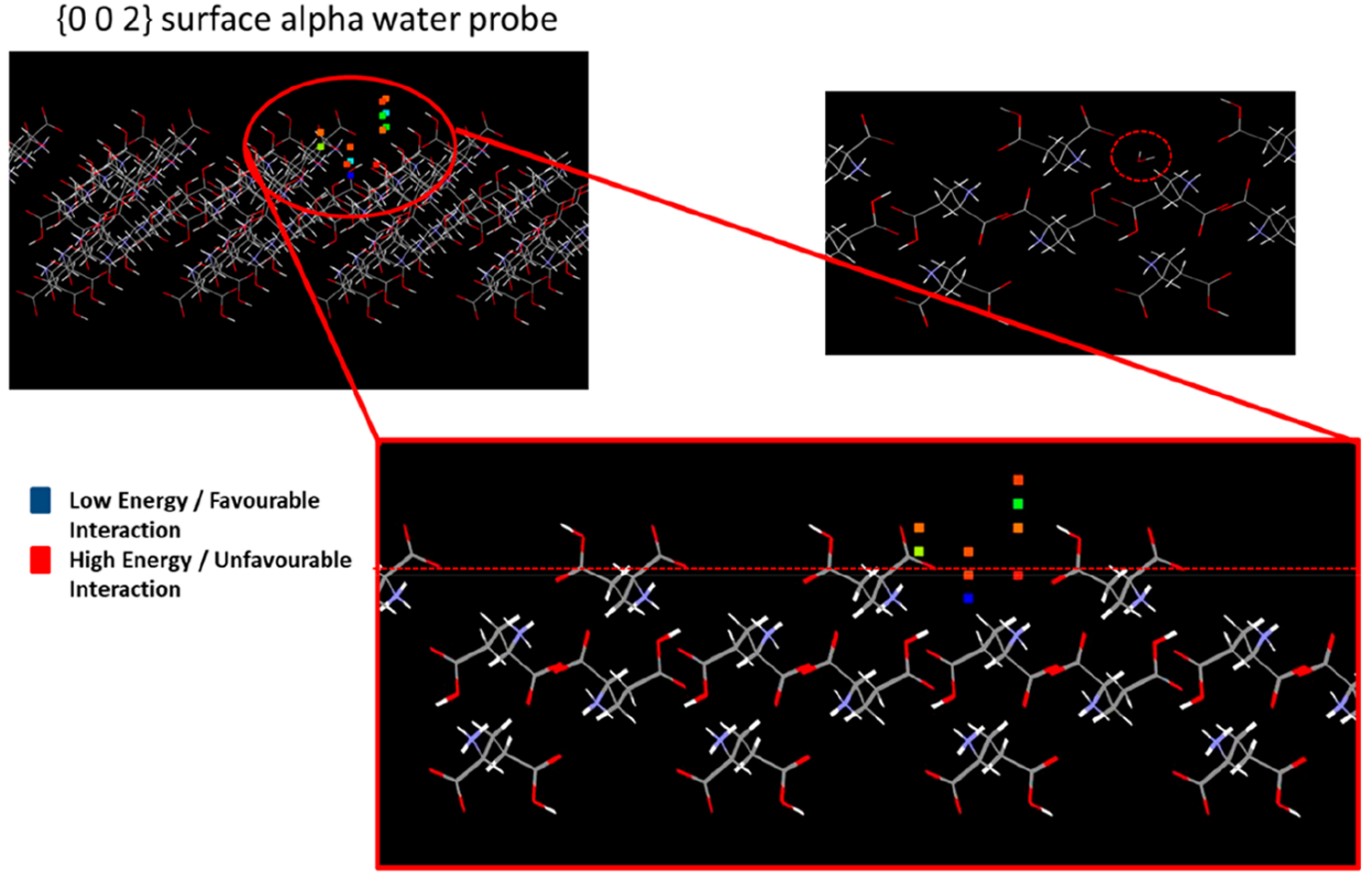
Surface search results highlighting the interaction locations of a water probe at the α {0 0 2} surface, indicating the surface topology and the surface “channels” where the most favorable interaction (blue square) is found for this surface.

**Figure 10:**
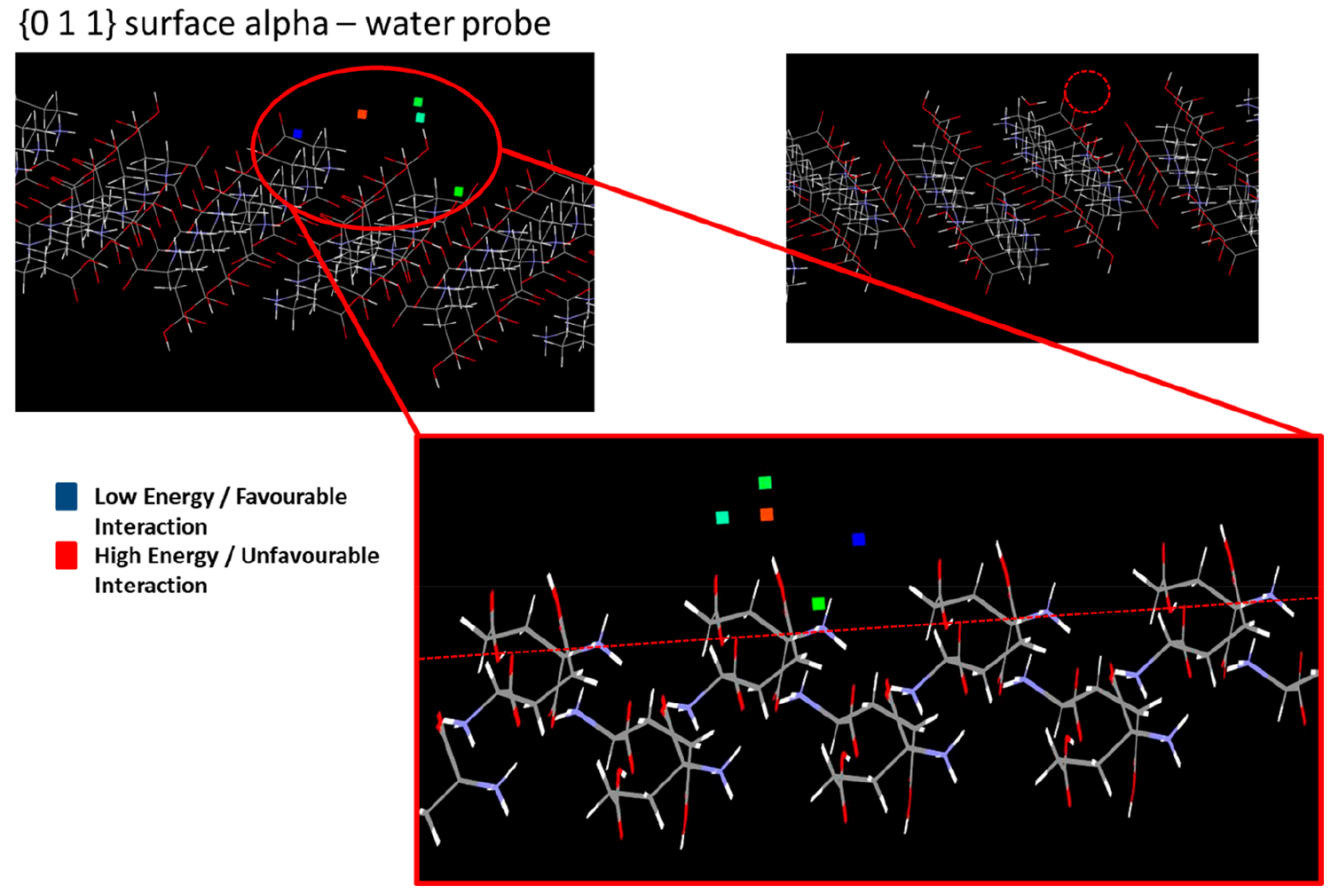
Surface search results highlighting the interaction locations of a water probe at the α {0 1 1} surface, indicating the relatively lower surface roughness compared to the {0 0 2} surface, and hence the lowest energy interaction (blue square) is located in a more surface accessible location.

Figure 9 and Figure 10 show representative examples of the grid-search results on the {0 0 2} and {0 1 1} surfaces of the α form. Figure 9 shows an example highlighting the interaction of a water probe molecule with the {0 0 2} surface, which is prevalent within the experimentally observed morphology of the α form. Here, the interactions are color-coded to highlight the strength of the interaction, with blue describing a favorable, and red the less favorable binding energies. Examination of the surface topology of the {0 0 2} surface also reveals it to exhibit a high degree of surface rugosity associated with a surface morphology at the molecular level which is characterized by channels which run along the *a* crystallographic axis. These channels contain a number of surface orientated acid and ammonium groups, which provide a strongly hydrophilic environment for the binding of solvation water. Unsurprisingly, the most favorable of the binding sites that were found (depicted as squares in the image) were found to lie within these surface channels (favorable interaction energy highlighted by the blue squares).

Figure 10 shows the result for water adsorption on the {0 1 1} surface, highlighting conversely that this surface has a much lower surface plane rugosity and one which is found to be characterized by a lack of hydrophilic channels. Consequently, the water probe molecules find their most energetically favorable surface-binding sites in more “accessible” locations, where they can bridge two acid groups, rather than being adsorbed within the surface microstructure as was the case with the {0 0 2} surface. Comparison between these two examples highlights not only the importance of the availability of solvent binding sites as a function of crystal habit faces but also the role played by surface rugosity, where rough crystal habit growth surfaces can impede solute and solvent mass transfer at the growth interface.

The situation for the β form polymorph is similar to that of the α form polymorph, where a visual analysis of two planes for the β form show that the {0 0 2} surfaces have a higher surface rugosity, enabling water to be trapped within surface channels which run along the surface. These channels contain the exposed ammonium groups of LGA which provide energetically favorable binding sites for water binding. Conversely, the {1 1 1} surfaces were found to have a lower plane rugosity than for the {0 0 2} surface and with the interaction field of the solvent at this surface being found to be more readily available for promoting desolvation. Detailed images related to surface search analysis of solvent water binding for the {1 1 1} and {0 0 2} surfaces are provided in Supporting Information S4.

#### 3.5.3 Examination of the Distribution of Surface Binding Sites and their Interaction Nature

**Figure 11:**
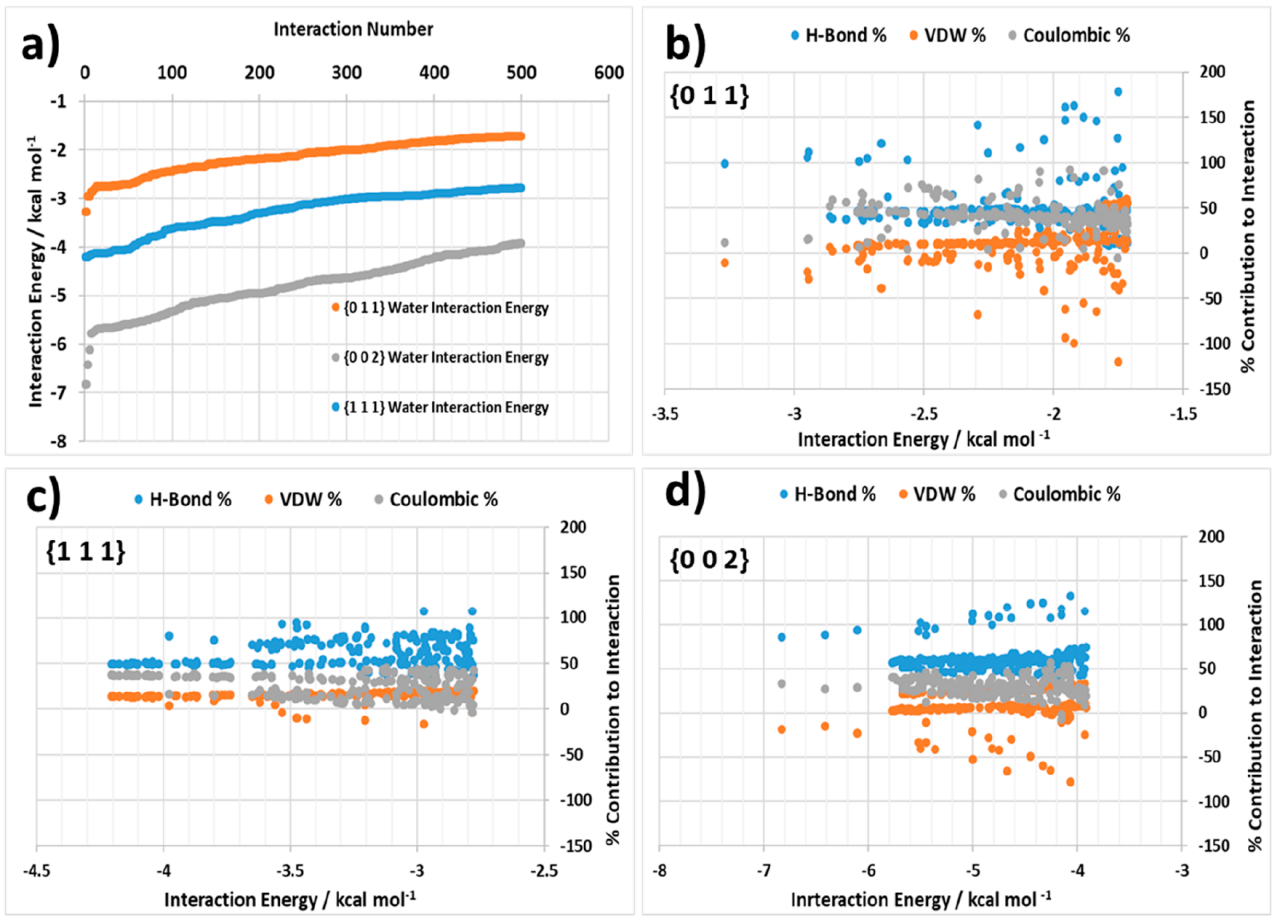
(a) Top 500 interactions ranked by the interaction energy of the water–surface binding for the α {0 1 1}, {0 0 2}, and {1 1 1} surfaces; (b) breakdown of the % contribution for the top 500 interactions of water with the {0 1 1} surface into H-bond, van der Waals, and Coulombic components of the interaction energy; (c) for the {1 1 1} surface and (d) for the {0 0 2} surface. Absolute energy plots of the same data are also provided in Supporting Information S5.

It is helpful also not just to examine the solvent binding sites with the highest interaction energies but also the distribution of energies as a function of the number of potential interactions. Figure 11 summarizes the results of examining the distribution of energies for the top 500 interactions using the surface search with water solvent probe molecules for the known experimentally observed crystal faces for the α form of LGA. The data, Figure 11a, show the binding energy profile for the three morphologically important crystal habit surfaces revealing a clearly delinerated separation profile for their water binding profiles with the most energetically favorable binding being at the {0 0 2} surface followed by the {1 1 1} surface and with the weakest binding being at the {0 1 1} surface; this reflects the trends in terms of the decreasing morphological importance of the habit planes for the experimentally grown single crystals. Figure 11b–d provides further detail concerning the % contribution of the three interaction types (van der Waals dispersive, H-bonding and Coulombic) that were probed through these simulations providing insight into the energy landscape for these interactions for the different habit surfaces. In this, the {0 0 2} and {1 1 1} surface interactions are found to be dominated by stronger H-bond and Coulombic interactions, whereas the interactions at the {0 1 1} surface were found to be more dispersive in nature overall.

**Figure 12:**
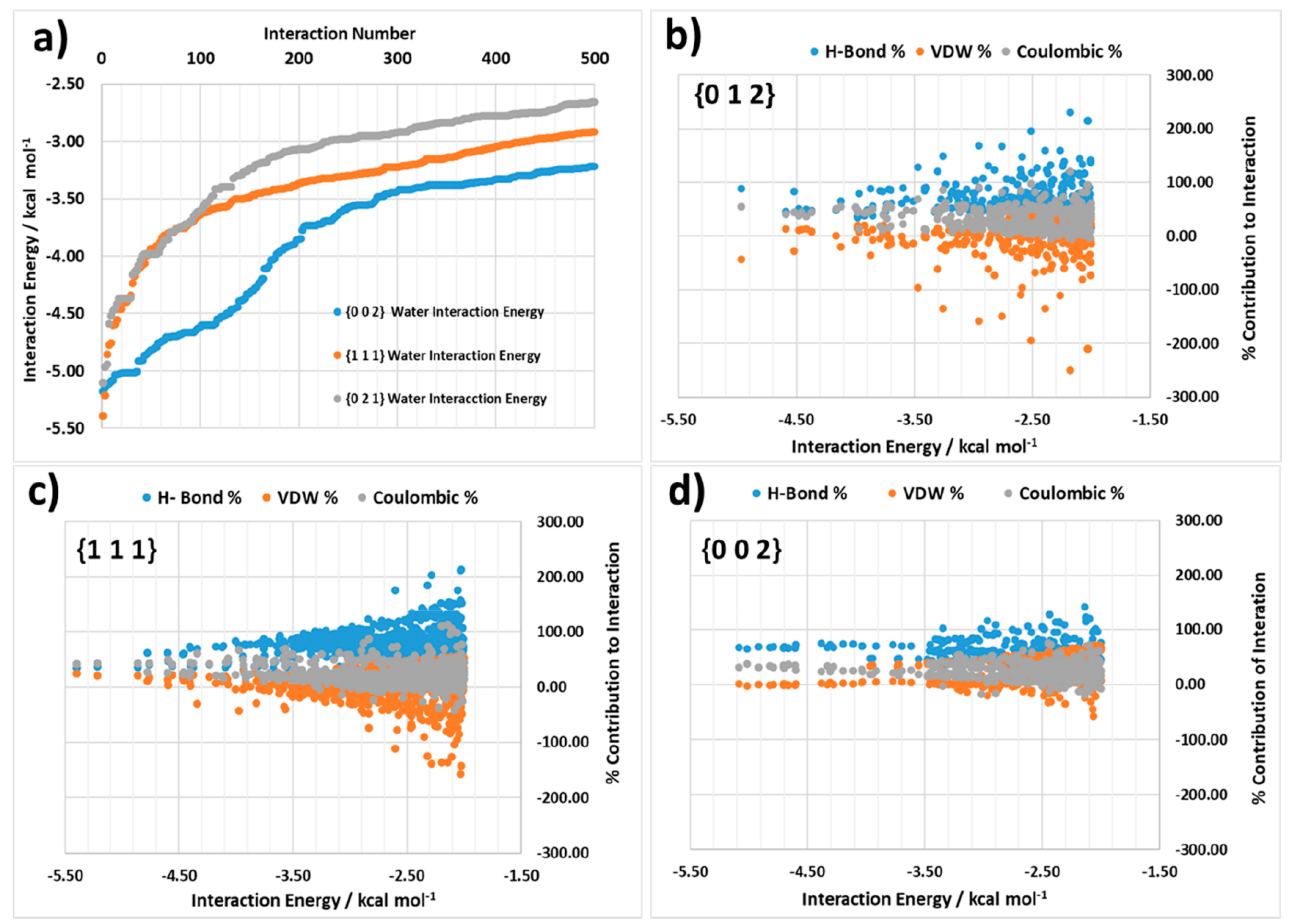
(a) Top 500 interactions ranked by interaction energy of the water–surface binding for the β {0 1 2}, {0 0 2}, and {1 1 1} surfaces, (b) breakdown of the % contribution for the top 500 interactions of water with the {0 1 2} surface into H-bond, van der Waals, and Coulombic components of the interaction energy, (c) for the {1 1 1} surface and (d) for the {0 0 2} surface. Absolute energy plots of the same data are also provided in Supporting Information S5.

Examination of the solvent-binding energies for the experimentally observed crystal faces of the β form of LGA are presented in Figure 12. Figure 12a shows that while the lowest interaction energies of water with the {0 0 2}, {1 1 1}, and {0 1 2} surfaces are quite similar, the interaction energies become more separated at lower interaction energies. This indicates that, overall, the interaction field of solvation binding sites for these three surfaces are quite similar in terms of their interaction energies when compared to the α form. Figure 12b–d highlights the contribution of the H-bond, van der Waals, and Coulombic contributions to the distribution of interactions for the {0 0 2}, {1 1 1}, and {0 1 2} surfaces. The data indicate no obvious differentiation between the interaction types for these three habit surfaces with all surfaces containing both strong H-bonding and Coulombic components.

### 3.6 Predicting the Solvent-Mediated Crystal Morphology

#### 3.6.1 Mechanistic Review of the Models

Overall, the analysis of the systematic search data for the crystal habit surfaces of the α and β forms using water and LGA as probe molecules highlights the importance of the surface topology of these surfaces in terms of how the solvent molecule interacts at these surfaces. The data indicate that a number of the morphologically important surfaces of these two polymorphs contain large channels with the potential for strong interactions with water through the exposed ammonium and carboxylate groups, which could lead to the formation of “trapped” solvation water within the surface regions.

This trapping of water would likely slow down the surface adsorption of LGA molecules associated with the growth of these surfaces, in particular, the {0 0 2} surface for the α and β form, during crystal growth. Conversely, the {0 1 1} surface of α form and the {1 1 1} surface of β form were found to have a lower plane rugosity, and as such the binding site of water was much more accessible. Additionally, the binding energy of water on these two surfaces was found to be much lower in comparison to the {0 0 2} surfaces of both forms. The combination of the two factors of rugosity and binding energy likely results in a higher rate of desolvation at these surfaces, which is much more favorable relative to the {0 0 2} surfaces, and hence it is likely the growth rate would be increased, which is in agreement with the experimental morphology for both polymorphs.

Overall, this leads to the postulate that consideration of surface topology can be important in predicting experimental face-specific crystal growth rates, particularly in terms of modeling the balance between surface desolvation and solute adsorption in directing crystal morphology. Considering this, the modified attachment energy model in eq 5 can be adapted to include a variation of surface topology through model integration with the calculated habit plane’s surface rugosity. Hence, the expression, eq 6, can be proposed for the calculation of *U*_*εhkl*__,_ where the surface plane rugosity, *R*_g_, is included as a fraction of the plane with the lowest rugosity, *R*_g min_.

While the above model provides a simple expression for the binding of solvent at crystal surfaces with varying surface rugosity, the binding of solute at the various surfaces is also impacted by the degree of solute binding sites as highlighted by Rosbottom et al.^5^ who through examination of solvent binding on the crystal habit surfaces of ibuprofen, highlighted the potential importance of the surface entropy α factor as a defining parameter for modeling both the crystal growth rate and its mechanism. This highlighted in particular its importance in modeling materials with anisotropic crystal morphologies such as needle-like and plate-like where the growth mechanism may vary significantly between slower and faster growing surfaces. The α factor calculation encompasses an assessment of the anisotropy of the extrinsic surface-terminated synthons and hence also provides an indication of surface roughening, i.e., transition from smooth to rough interfacial crystal growth. The α factor can be incorporated into a modified attachment model by considering the impact of this parameter upon the solute–surface interaction energy *U*_solute_, where a lower α factor is broadly consistent with a more uncontrolled growth process which provides a lower energy barrier to solute transport and integration at the growth interface. Hence, the ratio of *U*_solute_ and α factor for a given crystal habit surface simply provides a measure of this integration process, and hence the expression, described in eq 7 can be used to take this factor into account.

Overall, some caution should be taken in the application of these mechanistic models mindful of a number of assumptions made when applying the attachment energy theory for morphological prediction notably:

- The “equivalent surface wetting” criteria, which assumes that the solid–liquid intermolecular interactions formed at the crystal surfaces are equivalent to those formed in the bulk solution^79^
- The “surface/bulk structure equivalence” criteria, which assumes that surface-terminated crystal surface structure is equal to that in the bulk crystal structure and that no surface relaxation occurs^79^
- The proportionality criteria, which assumes that the strength of the various intermolecular interactions formed during crystal growth and dissolution processes; i.e., solid–solid, solid–liquid, and liquid–liquid interactions are in the same ratio for all the crystallographically independent crystal faces.^79^

#### 3.6.2 Rationalization of Models with Experimental Data

**Figure 13:**
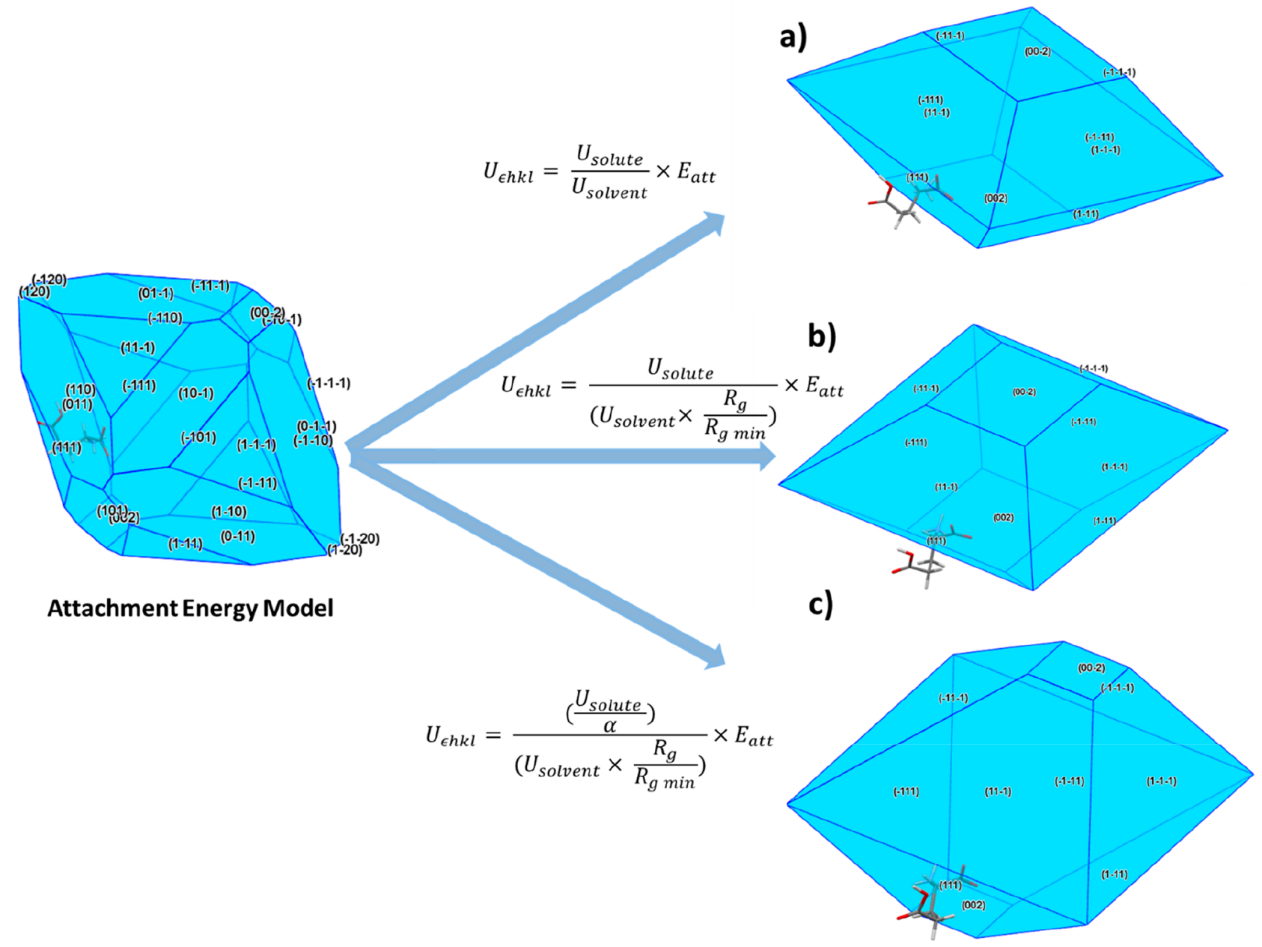
(a) Proposed models for calculating the α particle morphology using combined attachment energy and grid-based surface search methods; the attachment energy model is provided together with the calculated morphology based on the (a) surface interaction energy of the solute/solvent probes, (b) as (a) with surface rugosity factor accounted for in the solvent binding, (c) as (b) with α factor to account for surface anisotropy.

**Figure 14:**
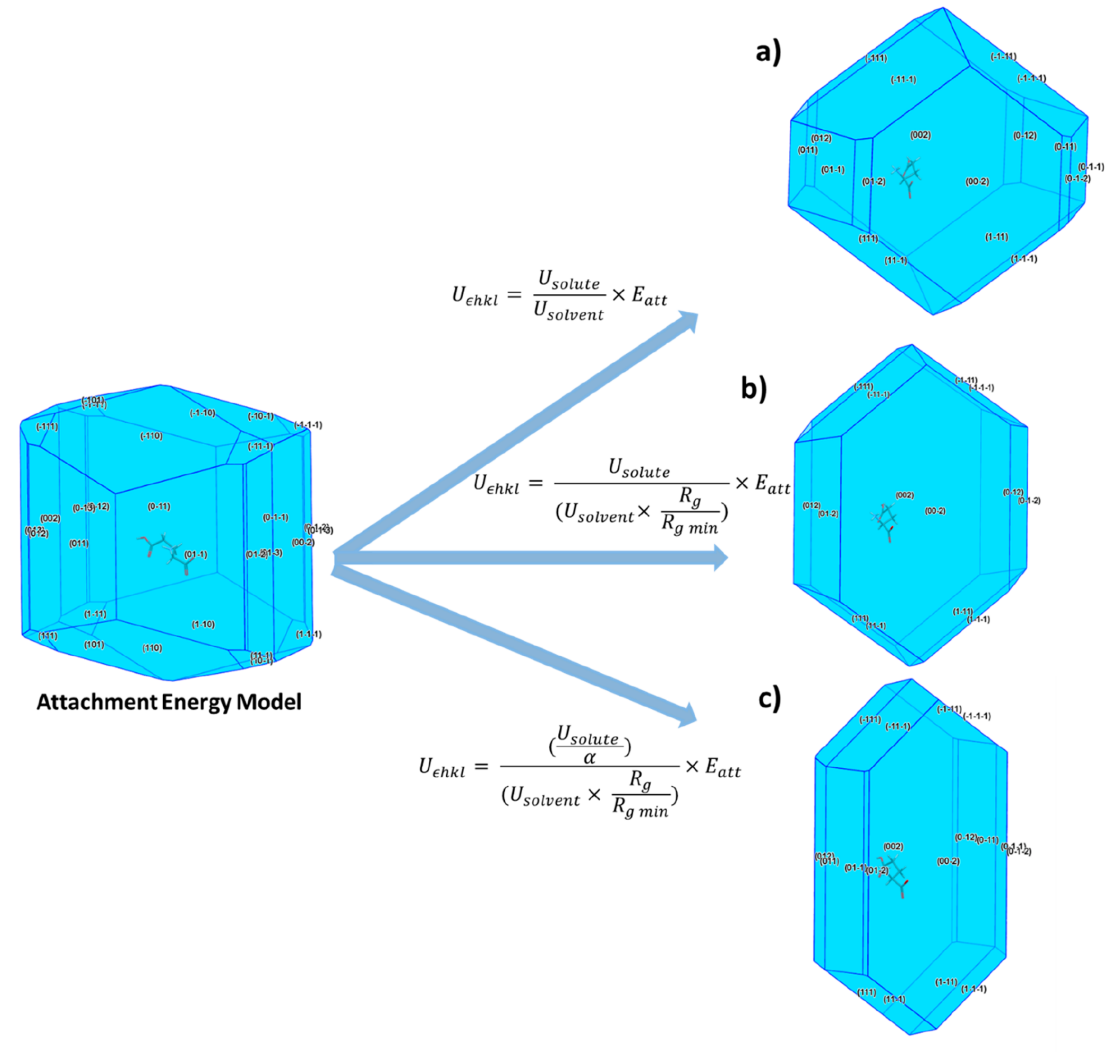
(a) Proposed models for calculating the β particle morphology using combined attachment energy and grid-based surface search methods; the attachment energy model is provided together with the calculated morphology based on the (a) surface interaction energy of the solute/solvent probes, (b) as (a) with surface rugosity factor accounted for in the solvent binding, (c) as (b) with α factor to account for surface anisotropy.

The three proposed models, described in eqs 5, 6, and 7 for morphology prediction were applied to the top eight planes of highest *d*-spacing for the α and β polymorphic forms and have been detailed in Table 3. The recalculated values of *U*_*εhkl*_ from the *E*_att_ terms were used as relative face-specific growth rates, and the final morphological shapes from the three models are provided for the α form in Figure 13 and for the β form in Figure 14.

The modified attachment energy models of the α form using the three models are dominated by the {1 1 1} and the {0 0 2} surfaces, which correlates well with SEM images of the α crystals grown from water solutions provided in Figure 7. The calculated morphology for the α-form was not found to differ greatly between the three proposed models, Figure 13a–c; however, the relative surface areas of the {0 0 2} and the {1 1 1} do change slightly. The model calculated from using eq 6, Figure 13a, provides the best agreement with the experimental morphology of the α-form crystals in Figure 7c,e.

The morphological models calculated for the β polymorph are provided in Figure 14a–c and provide an improvement for the morphological prediction using the modified attachment energy model for the observed needle-like crystals obtained from water solutions. The model provided in Figure 14 c provides the best correlation to the experimental morphology as shown in Figure 7d,f, where the particle morphology is dominated by the {0 0 2} surface, and the {1 1 1} faces are the needle-capping surface. The model also predicts the side faces to be a combination of {0 1 2} and {0 1 1} facets; however, it is difficult to determine if this is the case as the experimentally obtained crystals often appear as shards with very small side facets which are not always clearly observable to be effectivley indexed.

The improvement of the aspect ratio prediction to a needle-like particle using model (c) is due to the incorporation of the α factors within the attachment energy model and allows the likely differences between the growth rate parameters for the more stable prismatic {0 0 2} faces when compared to the less stable end-capping faces. Overall, the model prediction in Figure 14c provides a more realistic calculation of the crystal morphology when compared to the attachment energy model; however, the aspect ratio of the predicted morphology does not quite match that of the experimentally observed morphology. This is likely due to a number of factors, primarily those which are highlighted in section 3.6.1 but also because the models proposed do not rigorously take into account the growth mediation effects of impurity incorporation or the defect density at growth interfaces, both of which can be important particularly in the case of growth at potentially unstable surfaces such as those encountered in the crystal morphology of the β form.

#### 3.6.3 Rationalization of the Surface Chemistry with Respect to the Observed Crystal Morphology

**Figure 15:**
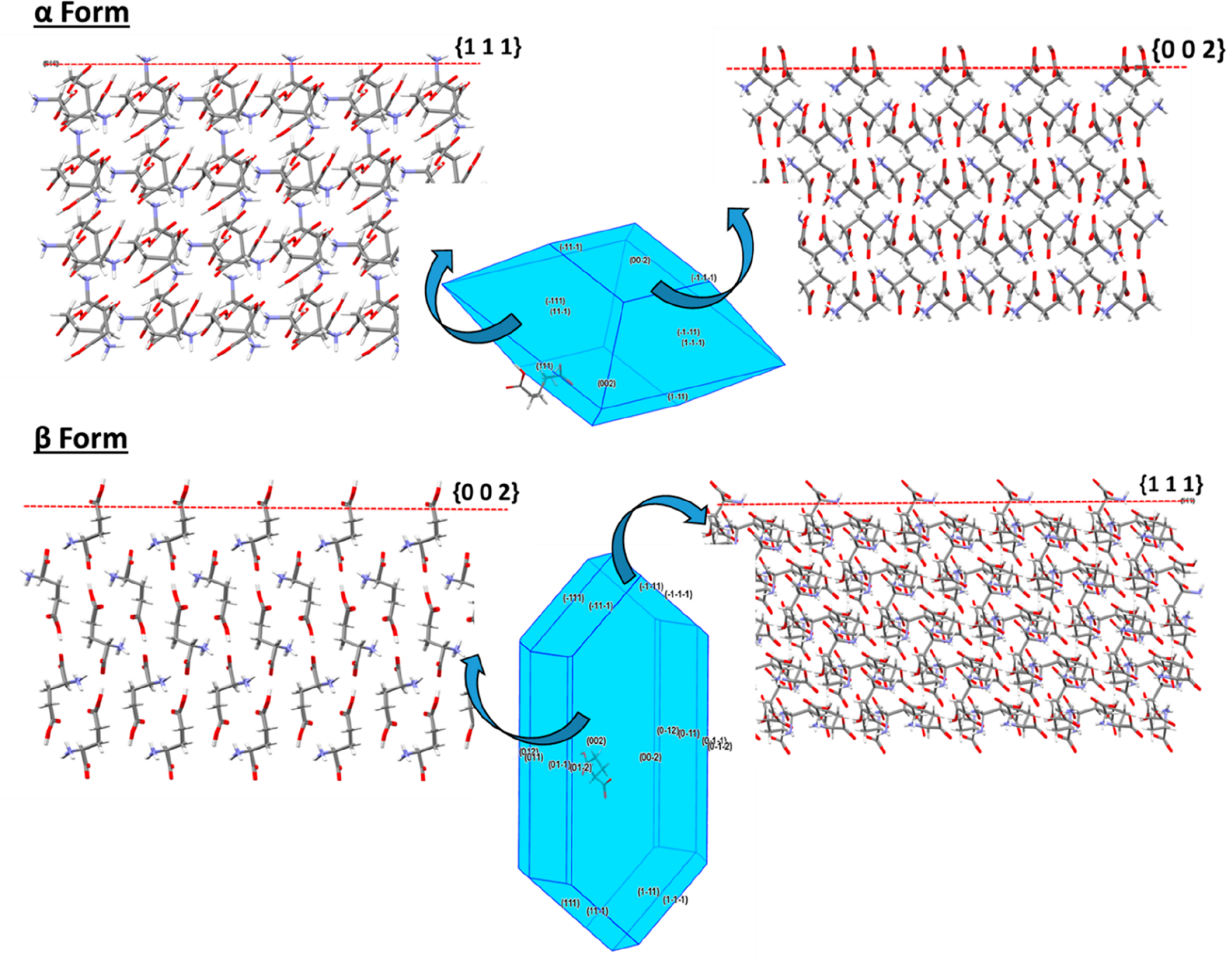
Morphological models of the α and β forms highlighting the surface chemistry of the slow-growing {0 0 2} surfaces in both forms and the {1 1 1} side facets of α and the capping {1 1 1} faces of the β needle morphology.

Identification of the surface-terminated extrinsic synthons for the observed crystal habit planes is summarized in Table 2 columns 9–12, and the morphologically important surfaces with their respective surface chemistry for the two polymorphs are highlighted in Figure 15. The data reveal that the α polymorph displays a quite isotropic contribution of its four energetically strongest synthons to the observed crystal habit where, interestingly, all synthons contribute a multiplicity of two to the *E*_att_ of all the observed habit faces, with the exception of synthon Aα to the {0 0 2} surface. The {0 0 2} surface often displays as a large flat habit face on the top of the prismatic morphology, indicating relatively slower growth than the other facets, as highlighted in the SEMs in Figure 7c,e. This correlates well with the synthon contribution to *E*_att_ at this surface, where the lack of the strong Coulombic synthon Aα, which has a relatively large intermolecular energy of −6.70 kcal mol^–1^ compared to Bα 4.32 kcal mol^–1^, could be expected to reduce the relative growth rate at this surface in comparison to the other habit faces. Overall, this partitioning of the synthons correlates well with the overall observed prismatic morphology of the α polymorph.

Analysis of the surface-terminated synthons for the β form highlights a contrasting surface chemistry landscape to that seen in the α form. Whereas in the α form the synthons were relatively isotropically distributed between the *E*_att_ contributions for the habit surfaces, in the β form the top four synthons are very anisotropically distributed between the three habit faces providing distinctively different surface chemistry of these faces. This is particularly obvious for the {0 0 2} surface for the β form where only the H-bonding synthon Cβ contributes positively to the crystal growth of this surface, while all other Coulombic synthons do not contribute at all. Conversely, portioning of the top four β polymorph synthons on to the {1 1 1} capping surface revealed that all the Coulombic interaction synthons Aβ, Bβ, and Dβ together with the H-bonding synthon Cβ contribute positively to the *E*_att_ at this surface. This highlights the dramatic difference in the broken bond energy which is available at the surfaces for growth in the β polymorph where the {1 1 1} capping surface has a range of available synthons for growth promotion, while the dominant {0 0 2} surface does not. This clear anisotropy of the β form surface synthons correlates well with experimental observations as evidenced by its anisotropic needle-like external morphology as highlighted in Figure 7d,f.

Overall, this analysis highlights the critical differences in terms of how the relative contributions of the bulk synthons found in the crystal structures for the two forms contribute quite differently to the surface growth process of the crystal habit faces found in the observed crystal morphologies. This, in turn, correlates very well with the isotropic and anisotropic nature of the structures and morphologies for the α and β forms, respectively, as well as their propensity for surface binding.

## 4 Conclusions

This work highlights the application of synthonic engineering approaches encompassing molecular-scale mechanistic models and associated workflows for the identification of the important solid-state and surface-terminated intermolecular synthons as a function of their interacting functional groups and energies in order to characterize and understand the physicochemical properties and crystallization behavior of materials. The data highlight how the lattice energy of polymorphic systems can be used to infer the relative stability between polymorphic forms, and further to this, how the specific intermolecular interactions which stabilize the crystal lattice can be identified and ranked through intermolecular synthonic modeling using the atom–atom summation method. The predicted crystal morphologies for the α and β forms of LGA have been correlated with their experimentally observed particle morphologies. The surface-specific intermolecular interactions of solute and solvent at lattice planes for both polymorphs were quantified using the systematic search methods. The work provides a quantitative understanding of the role played by solvent and surface topology in directing the observed crystal morphologies when crystallized from aqueous solution. The predicted solvent-dependent morphologies were assessed through a number of mechanistic model predictions for the α polymorph and were found to be in very good agreement with the experimentally observed morphology, while those for the β morphological model provided a fair representation of the needle-like experimental morphology. The observed crystal morphologies and associated surface chemistry were also rationalized through characterization of the surface-terminated bulk synthons at the external habit surfaces. The α form was found to have a very isotropic synthon contribution to the morphological surfaces, whereas the β form had a much more anisotropic contribution, predictions which correlated well with their experimentally observed crystal morphologies.

This solvent-mediated morphological prediction model has been developed into a digital mechanistic workflow for the routine modeling of these effects at the point of the crystallization process R&D. Overall, this work has demonstrated the utility of a predictive model for the solvent-dependent morphology of organic materials from their root crystallographic structures through the application of mechanistically based workflows encompassing solvent binding, surface interaction energies, and surface rugosity.

## Supporting Information Available

Detailed overview of the workflow developed as part of this study for modeling solvent-dependent morphology (S1), together with detailed molecular diagrams of the intermolecular synthons for the two polymorphs with a table of their energy breakdown (S2+S3). Additionally, detailed figures highlighting the surface search results of water binding on various habit planes of the two polymorphs is also provided together with plots of the surface search quantitative breakdown of the surface binding energy (S4+S5) (PDF)

## Dedication

Dedicated to the life and works of Professor Roger J Davey.

## List of Symbols

*E*_latt_: lattice energy, kcal mol^–1^
*E*_sl_: slice energy, kcal mol^–1^
*E*_att_: attachment energy, kcal mol^–1^
*ΔE*_pt_: proton transfer energy, kcal mol^–1^
*ε*_*hkl*_: anisotropy factor
*ΔH*_s_: sublimation enthalpy, kcal mol^–1^
*ΔH*_f_: enthalpy of fusion, kcal mol^–1^
*R*_g_: surface rugosity, Å
*U*_*εhkl*_: modified attachment energy of specific *hkl* plane
*U*_solute_: interaction energy of solute on surface, kcal mol^–1^
*U*_solvent_: interaction energy of solvent on surface, kcal mol^–1^
*X*_seq_: mole fraction of solute
*Z*: number of formula units within the crystallographic unit cell
*Z*′: number of formular units in the asymmetric unit
