# Supplementary material for: A Digital Mechanistic Workflow for Predicting Solvent-Mediated Crystal Morphology: The α and β Forms of l-Glutamic Acid
Source: Cryst Growth Des. 2022 Apr 11;22(5):3042–59. doi: 10.1021/acs.cgd.1c01490 (PMC9073950; doi:10.1021/acs.cgd.1c01490)
Supplement: Supplementary file 1 — cg1c01490_si_001.pdf [file cg1c01490_si_001.pdf]

Supporting Information

A Digital Mechanistic Workflow for Predicting Solvent-Mediated Crystal Morphology: The α and β Forms of L-Glutamic Acid

Thomas D. Turner*[1+], Neil Dawson[2], Martin Edwards[3], Jonathan H. Pickering[1$], Robert B. Hammond[1], Robert Docherty[2] and Kevin J. Roberts[1]

[1] Centre for the Digital Design of Drug Products, School of Chemical and Process Engineering, University of Leeds, Woodhouse Lane, Leeds, LS2 9JT

[2] Pfizer R&D Ltd, Ramsgate Road, Sandwich, Kent, CT13 9NJ

[3]Britest Limited, Keckwick Lane, Daresbury, Warrington WA4 4FS

Current Address:

+School of Chemistry, University of Leeds, Woodhouse Lane, Leeds, LS2 9JT

$School of Computing, University of Leeds, Woodhouse Lane, Leeds, LS2 9JT

** Corresponding Author: t.d.turner@leeds.ac.uk*

Dedicated to the life and works of Professor Roger J Davey

Keywords

Crystallisation, solvent-mediated crystal habit modification, crystal morphology and surface chemistry, molecular and synthonic modelling of inter-molecular interactions, polymorphism, surface roughening, lattice energy, L-glutamic acid

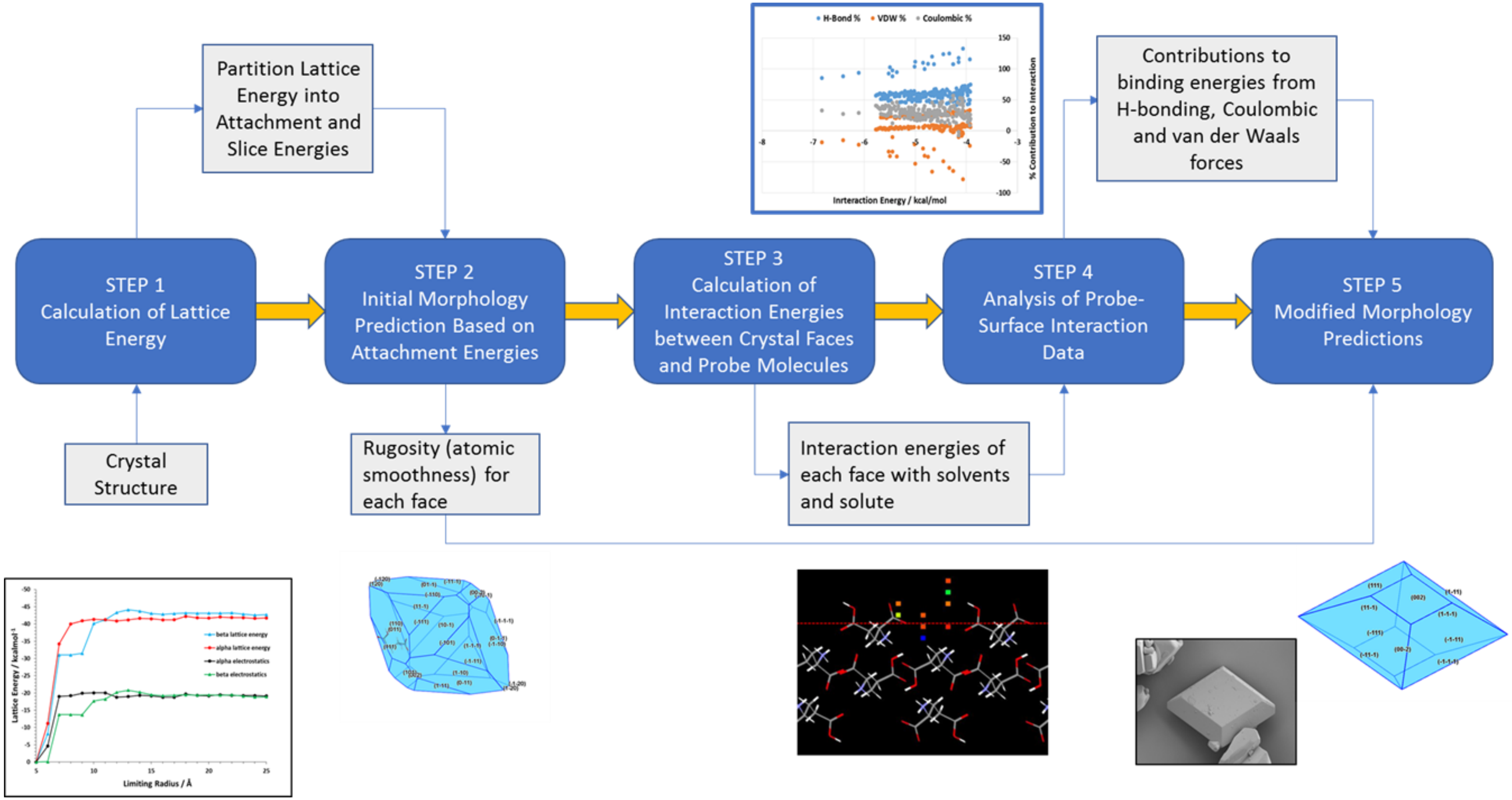

Figure S1 High-level 5 step process workflow for the prediction of solvent dependent particle morphology using molecular and crystallographic modelling tools.

S1. Solvent Dependent Morphology Workflow

The workflow, outlined in Figure 1, can be broadly defined by five major steps:

i. Calculation of the materials lattice energy through an atom-atom summation method using a user defined forcefield and a crystallographic information structure file (CIF).
ii. Partitioning of the lattice energy to slice and attachment energy of the top 10 BFDH (Bravais, Friedel, Donnay, Harker) crystal planes ranked from largest interplanar d-spacing (#1) to the lowest interplanar d-spacing (#10), to yield the crystals' attachment energy morphology model. Surface anisotropy factors and plane rugosity as a function of hkl are also calculated in this step.
iii. Calculation of the interaction energies of probe solute and solvent molecules at crystallographic planes (user defined, but usually top 10 BFDH list as defined in step II), through an atom-atom summation calculation and a user defined forcefield (user defined, but usually same as step I)
iv. Analysis of the interfacial chemistry of the interaction field at the various surfaces to provide a breakdown of dispersive, H-bonding and coulombic type interactions. The simulation provides a visual representation of this interaction energy field as a user check for the calculation – edge effects, probe to surface distances etc. Additionally, visual identification of the lowest energy (most favourable) probe-surface binding site is achieved through this step. Any calculation discrepancies identified in step iv results in calculation optimisation through repetition of step iii. Once optimised data is collected the energies of the most favourable binding sites for solvent and solute are tabulated as a function of crystal plane.
v. Modified attachment energy morphology based on the solute / solvent surface binding ratio, plane rugosity and surface reactivity

A detailed description of this workflow is provided in following section.

Step I. Lattice Energy Calculation

The workflow begins with loading the crystal structure of the molecule of interest, followed by initial checks of the atom and bond types in the structure with any required atom or bond amendments processed at this point. The lattice energy, E_{cr}, of the material is then calculated using an atom-atom summation method which calculates the intermolecular interactions of a central molecule in the unit cell with all other molecules within a defined sphere of surrounding unit cells. This sphere can be spatially extended up to a user defined distance to assess the lattice energy convergence. The interatomic interactions are calculated through a user defined forcefield and electrostatic potential energies are calculated using charges from the Gasteiger method.

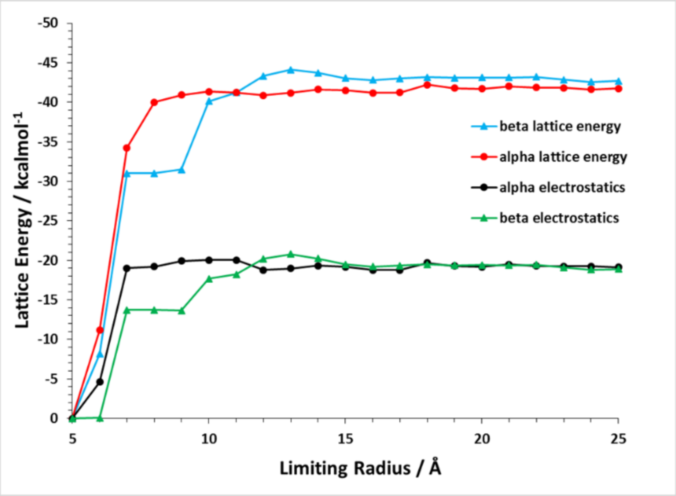

Step II. Attachment Energy Morphology Calculation

Following the calculation of the E_{cr} the most likely growth slices are selected by ranking the lattice planes as a function of decreasing d-spacing where those with largest d-spacing are selected for further analysis. For these slices the E_{cr} is partitioned into slice, E_{sl}, and attachment energy, E_{att}. The relative E_{att} are then expressed as centre to face distances to calculate an E_{att} morphology through the relative E_{att} growth rate approximation.

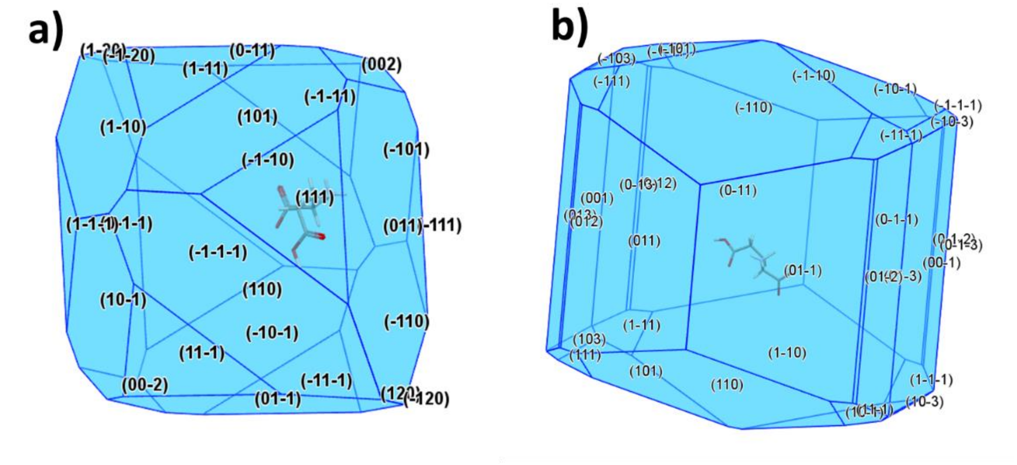

The slices selected through the d-spacing ranking rules are then analysed in terms of their surface anisotropy, ε_{hkl}, which describes the surface saturation of synthons (inter-molecular interactions) for a defined surface upon termination of that surface. This is a measure as to the surface 'reactivity', and a larger value reflects a surface with a larger proportion of its synthons chemically satisfied and hence is less reactive. This parameter is also related to the *α* factor of a particular surface through the following expression

$$\alpha = \varepsilon_{hkl}\left(\frac{\Delta H_f}{RT} - \ln X_{seq}\right) \tag{2.12}$$

where ΔH_f is the heat of fusion and X_{seq} is the mole fraction of the solute at a relevant supersaturation and temperature for crystal growth of the solute system, *R* is the gas constant and *T* is temperature. The *α* factor describes the growth mechanism by which a crystal surface is likely to grow where $\alpha < 2$ is likely to yield rough interfacial growth, $2 < \alpha < 5$ is likely to grow through a birth and spread model and $\alpha > 5$ is likely to be growth via a screw dislocation mechanism. The plane rugosity, R_g, of the selected planes is then calculated, where R_g represents the atomic smoothness of each plane in Å and hence a larger value represents a 'rougher' surface at the atomic scale.

Step III. Calculation of Probe – Surface Interaction Energy

The selected slices are then analysed in terms of their interactions with two relevant probe molecules using the systematic search tool, the first is a probe molecule of the

solute of interest and the second is the molecule of solvent for which the solvent dependent morphology is to be calculated.

The desired surface of crystal is built within systematic search and the user defines a grid which will sit on the crystal surface. During the computational procedure the probe molecule is placed at each position on the grid where at each position the probe is rotated about three Euler angles and at each rotation the interaction energy between the probe and the surface. The interaction energy is calculated through a rigid body, atom-atom summation method with no surface relaxation, where a user defined atomic force field in combination with atom charging by the Gasteiger method is used.

0 0 2 surface alpha water probe

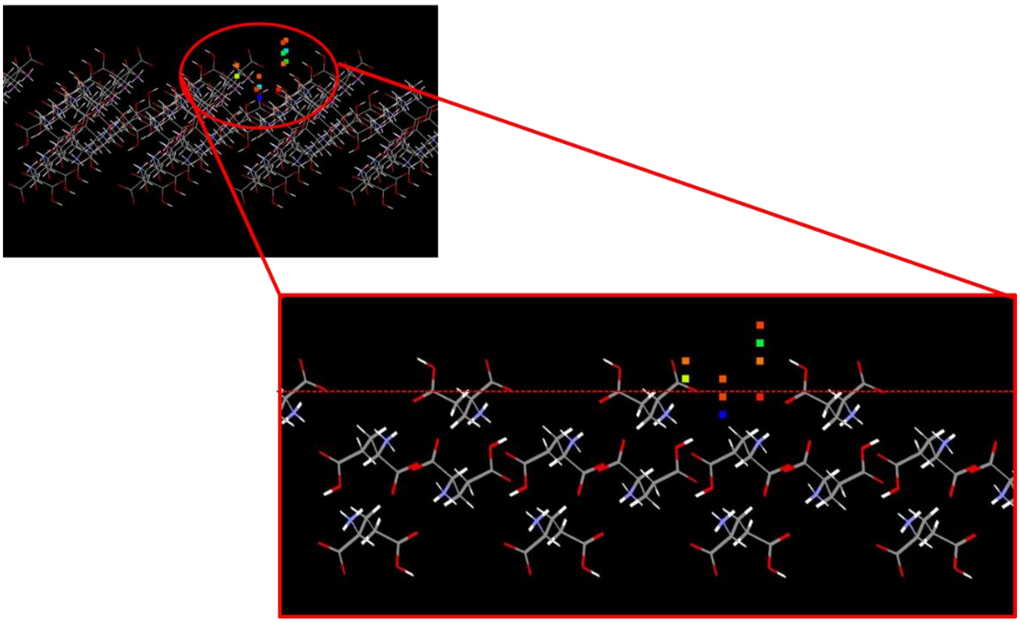

Step IV. Analysis of Probe-Surface Interaction Energy Data

The interaction energy field of the probe molecule with the surface is outputted as a data file where interactions which pass a user defined energy filter are captured and the interactions are ranked as a function of total energy in kcal / mol. The systematic search tool also allows the user to view interactions visually highlighting a coloured grid which informs the user as to the strength of the interactions at that grid point. Additionally, specific interactions may be visualised showing the probe position relative to the surface.

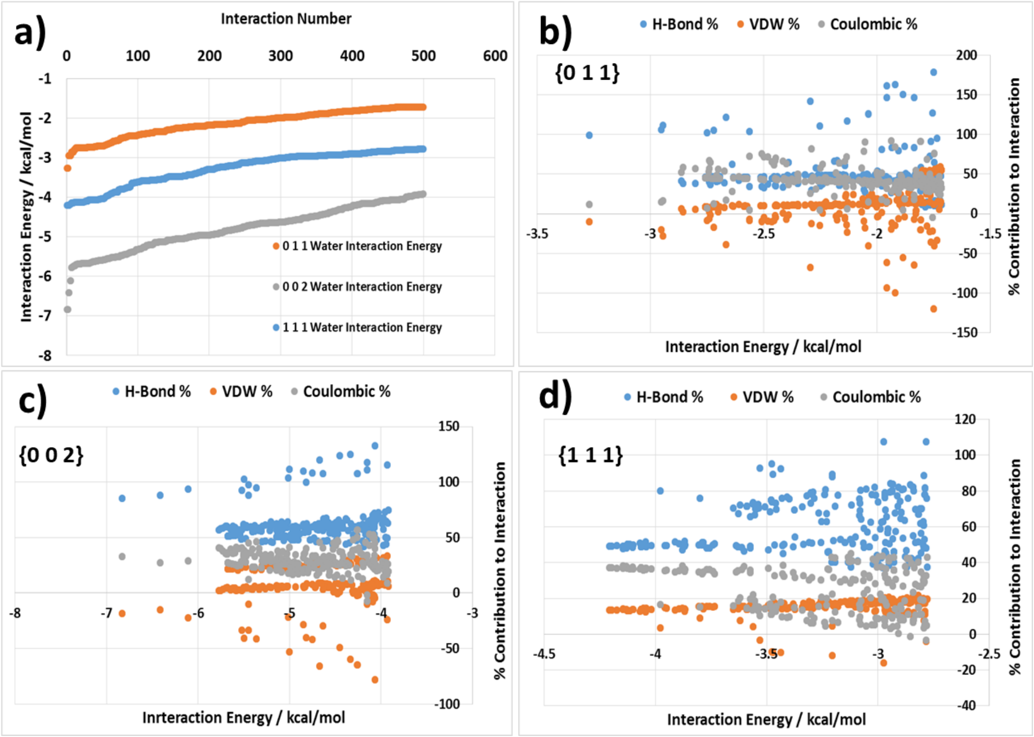

The lowest interaction energy (most favourable) for the solute, U_{solute}, and solvent, $U_{solvent}$, surface interactions is then analysed as a function of the selected slices chosen in Step II and the ratio of these low energy interactions is recorded for each slice. Further to this the chemistry of these interactions is also contained in the output file highlighting the contribution of dispersive, H-bonding and coulombic type interaction energy to the total values.

Step V. Calculation of Modified Attachment Energy Morphology for Solvent Dependency

Finally, the calculated data including values of, R_g, α, U_{solute} / $U_{solvent}$ and E_{att} for each slice is then used to describe the relative E_{att} growth rate approximation through the models described in expressions 2.13, 2.14 and 2.15.

$$U_{\epsilon hkl} = \frac{U_{solute}}{U_{solvent}} \times E_{att} \quad (2.13)$$

$$U_{\epsilon hkl} = \frac{U_{solute}}{(U_{solvent} \times \frac{R_g}{R_{g\,min}})} \times E_{att} \quad (2.14)$$

$$U_{\epsilon hkl} = \frac{(\frac{U_{solute}}{\alpha})}{(U_{solvent} \times \frac{R_g}{R_{g\,min}})} \times E_{att} \quad (2.15)$$

These expressions use the data calculated to describe the growth of specific planes within a crystal to form its external morphology. The first model, 2.13, describes the balance between relative binding energies of the solute versus the solvent at a specific surface, this is to represent the driving force for integration of a solute molecule contrasted with de-solvation of that surface during the growth process. The second model, 2.14, has the addition of a term which describes the rugosity of the surface and corrects the solvent binding energy based on how atomically smooth a surface is. This is to account for the steric hindrance of removing a molecule of solvent from the surface when an increase number of low energy binding sites are located in surface channels. Hence a rougher surface will be more difficult to de-solvate than a smooth surface and this term corrects the binding energy accordingly. The final model, 2.15, includes a term to account for the face-specific growth mechanism expected to occur at that surface based on the anisotropy (broken bond energy) calculated at the surface. This term aims to correct the solute binding energy based on the likely mechanism of integration of solute molecules at the surface and is particularly important for growth of unstable surfaces which generally result in a needle-like particle morphology.

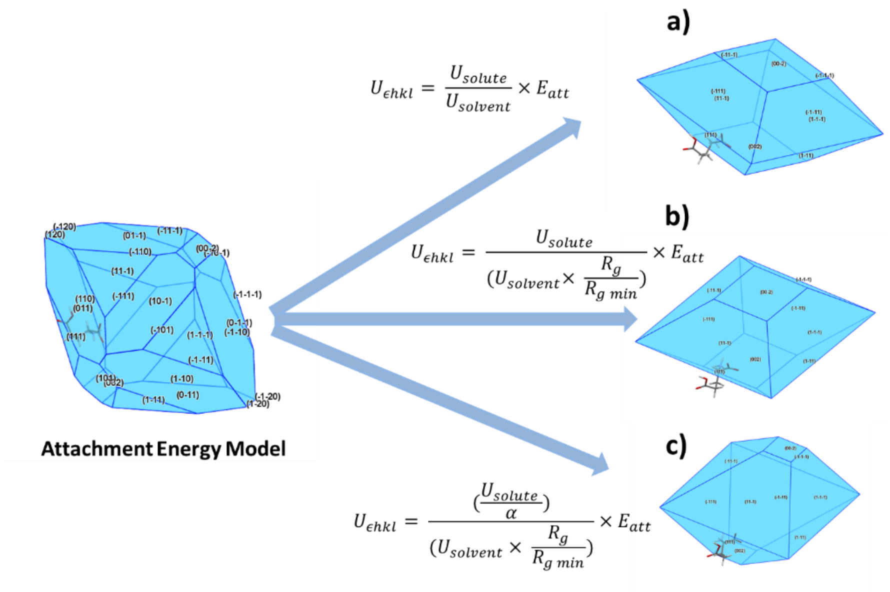

S2. Detailed Molecular Diagrams of Intermolecular Synthons

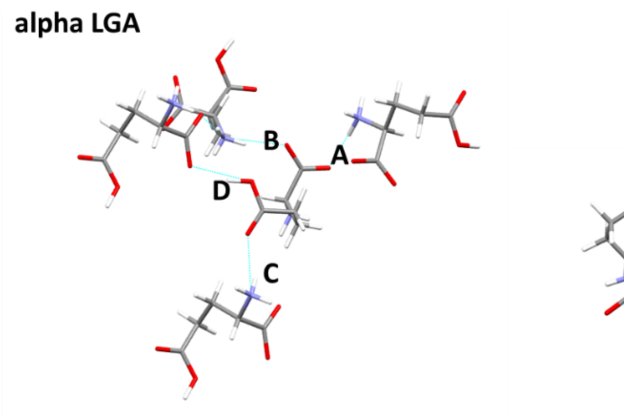

Bond	Multiplicity	*Distance (Å)	Intermolecular energy(kcal mol^{-1})	%contribution to lattice energy
A	2	6.18	-6.70	32.03
B	2	5.64	-4.32	20.66
C	2	5.53	-3.75	17.93
D	2	7.19	-3.04	14.54
				**Total 85.15**

*centre of mass distances

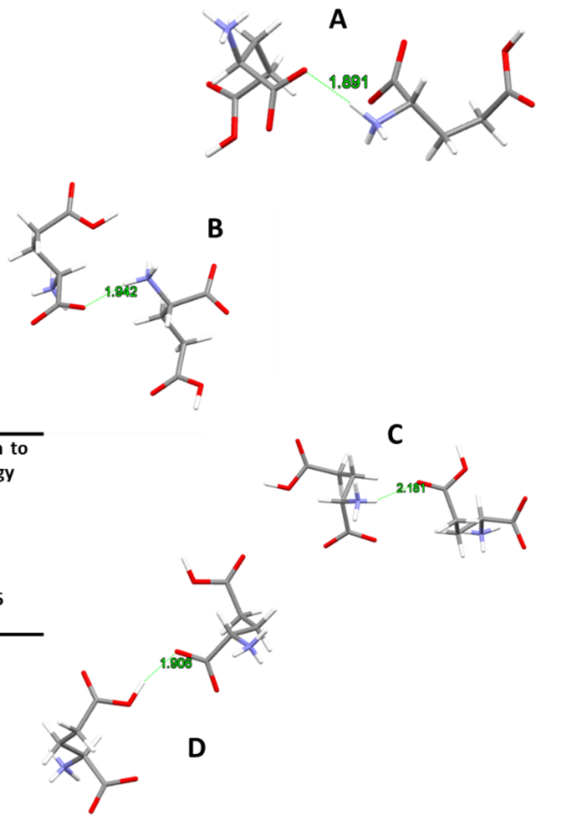

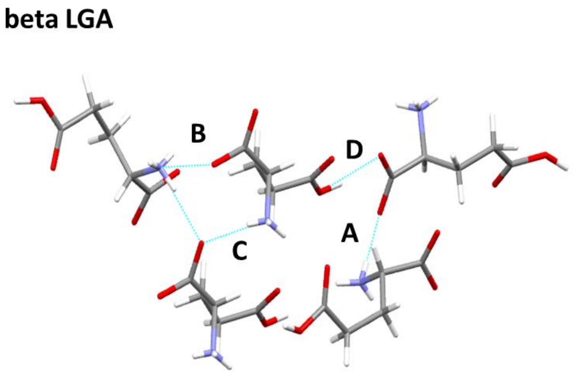

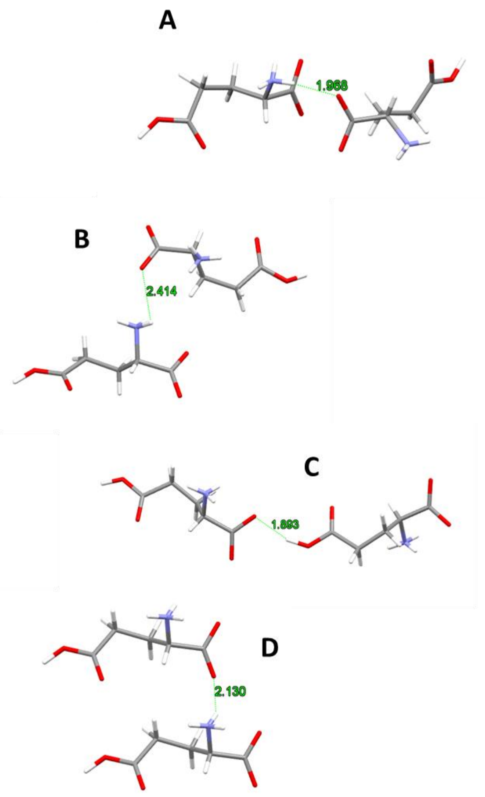

Bond	Multiplicity	*Distance(Å)	Intermolecular energy(kcal mol^{-1})	% contribution to lattice energy
A	2	6.23	-5.88	27.33
B	2	6.07	-5.36	24.91
C	2	9	-4.14	19.24
D	2	4.96	-2.25	10.46
				Total 81.94

*centre of mass distances

S3. Table of Intermolecular Synthon Energy with Interaction Type Breakdown

Bond	Multiplicity	Distance(Å)*	Intermolecular energy(kcal mol^{-1})	Dispersive Total (kcalmol^{-1})	Coulombic Energy (kcal mol^{-1})	% Contribution to E_{latt}	Intermolecular Interaction Type
α polymorph							
Aα	2	6.18	-6.70	-1.67	-5.02	32.03	COO$^-$…NH$_3^+$ **Coulombic**
Bα	2	5.64	-4.32	-2.12	-2.2	20.66	COO$^-$…NH$_3^+$ **Coulombic**
Cα	2	5.53	-3.75	-1.39	-2.36	17.93	NH$_3^+$…O= **H-bond**
Dα	2	7.19	-3.04	-2.17	-0.87	14.54	OH…COO$^-$ **H-bond**
β polymorph						**Total 85.15**	
Aβ	2	6.23	-5.88	-2.14	-3.73	27.33	COO$^-$…NH$_3^+$ **Coulombic**
Bβ	2	6.07	-5.36	-1.07	-4.29	24.91	COO$^-$…NH$_3^+$ **Coulombic**
Cβ	2	9.00	-4.14	-2.01	-2.12	19.24	OH…COO$^-$ **H-bond**
Dβ	2	4.96	-2.25	-2.03	-0.22	10.46	COO$^-$…NH$_3^+$ **Coulombic**
						**Total 81.94**	

S4. Surface Search Data for Beta Polymorph

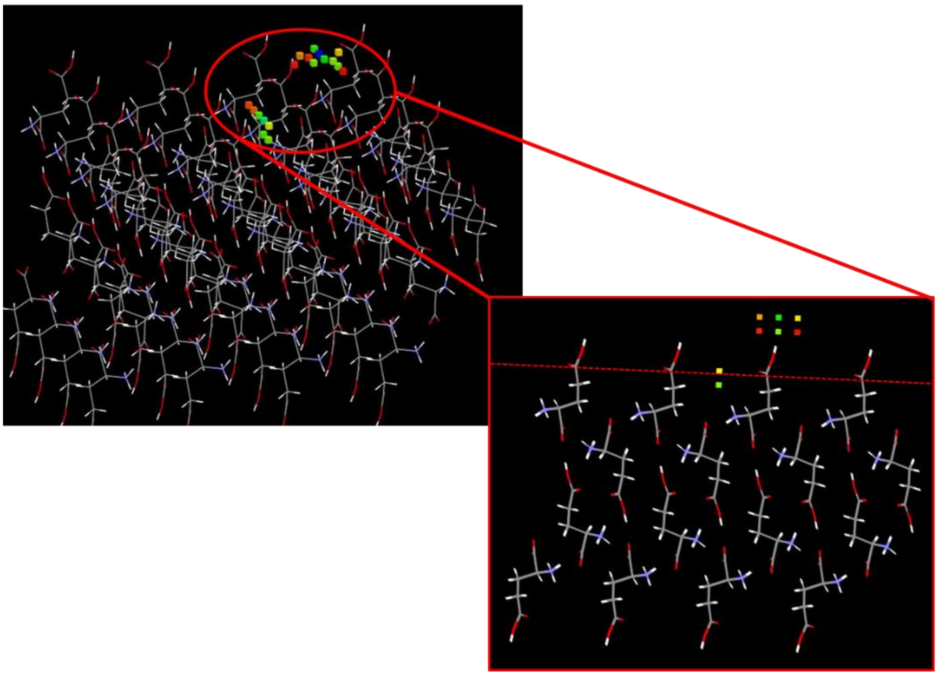

Figure S2. Surface search results highlighting the interaction locations of a water probe at the β {0 0 2} surface and the high plane rugosity of the surface which provides low energy binding sites for the solvent (green and yellow squares) in a similar manner to the α form.

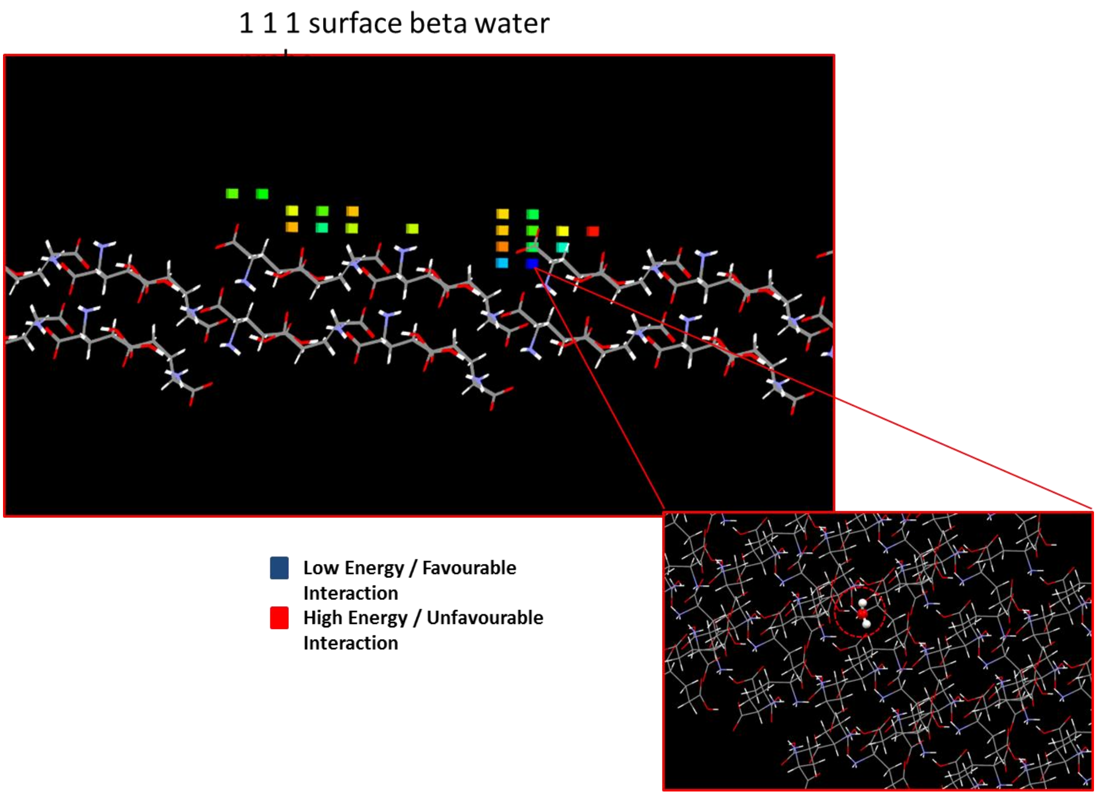

Figure S3. Surface search results highlighting the interaction locations of a water probe at the β {1 1 1} surface and the observed low plane rugosity of the surface, in comparison to the {0 0 2} surface the lower energy binding sites are more surface ‘accessible’ by solute molecules.

S5. Surface Search Data of Top 500 Interactions Plotted vs Energy

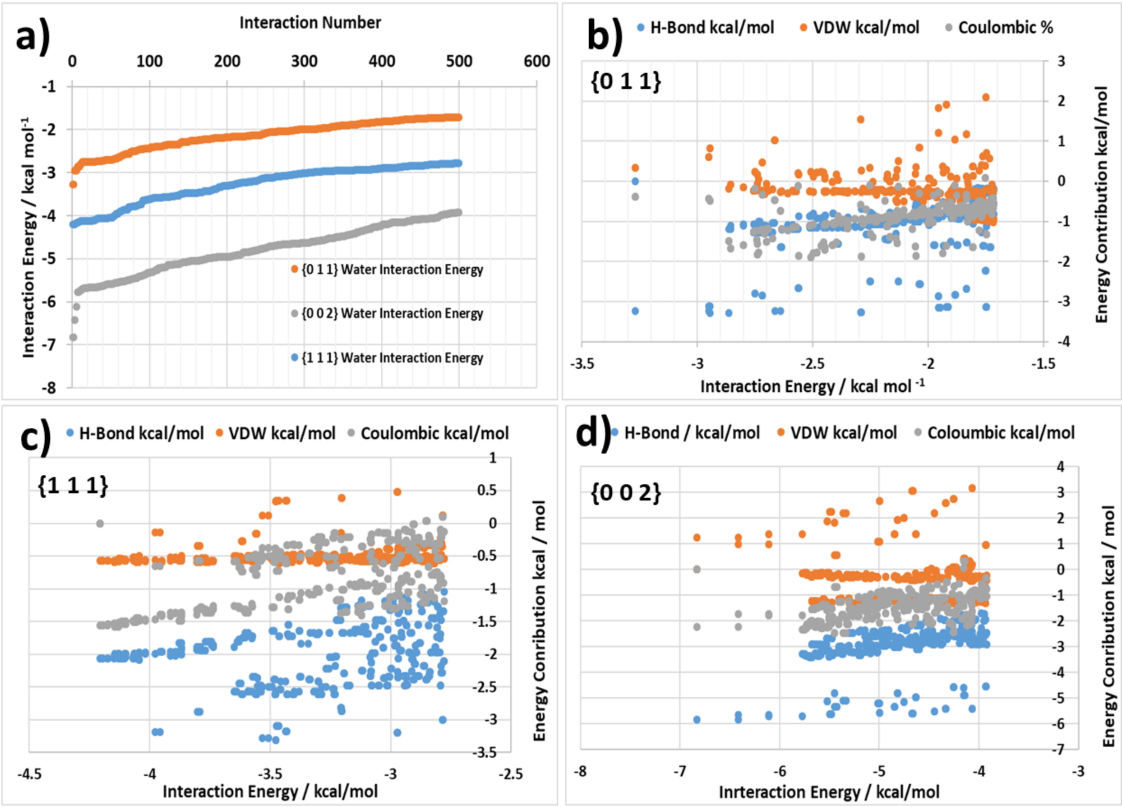

Figure S4 a) top 500 interactions ranked by interaction energy of the water – surface binding for the α {0 1 1}, {0 0 2} and {1 1 1} surfaces, b) breakdown of the absolute energy contribution for the top 500 interactions of water with the {0 1 1} surface into H-bond, van der Waals, and coulombic components of the interaction energy, c) for the {1 1 1} surface and d) for the {0 0 2} surface.

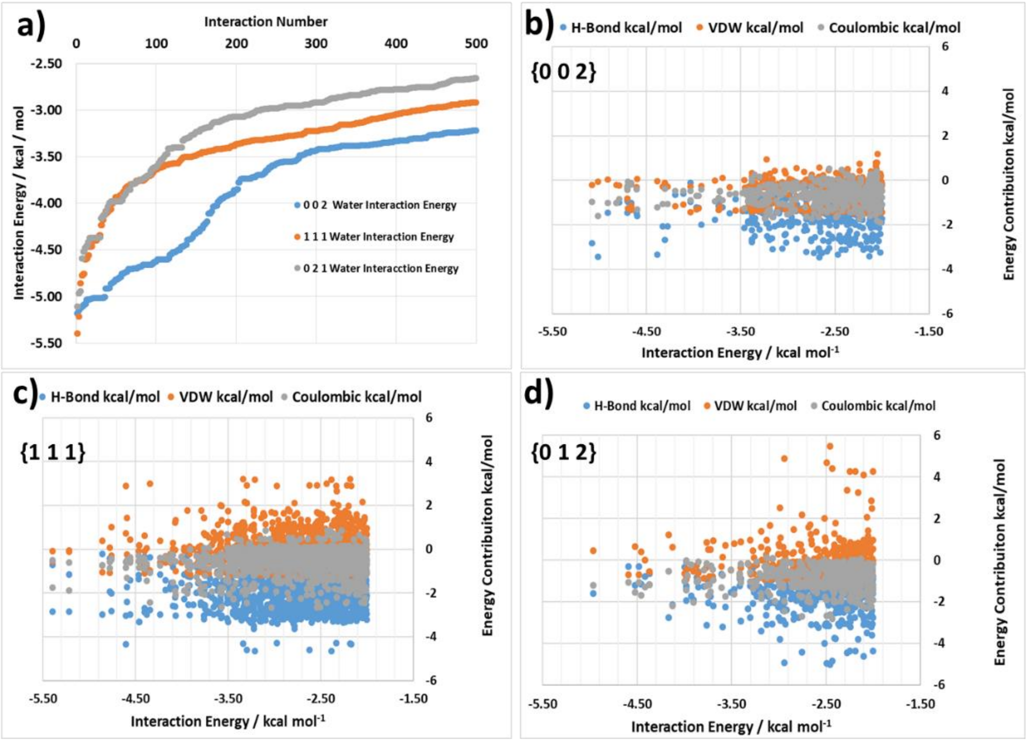

Figure S5 a) top 500 interactions ranked by interaction energy of the water – surface binding for the β {0 1 2}, {0 0 2} and {1 1 1} surfaces, b) breakdown of the absolute energy contributions for the top 500 interactions of water with the {0 0 2} surface into H-bond, van der Waals, and coulombic components of the interaction energy, c) for the {1 1 1} surface and d) for the {0 1 2} surface.
